# Pectin-based bioinks for 3D models of neural tissue produced by a pH-controlled kinetics

**DOI:** 10.3389/fbioe.2022.1032542

**Published:** 2022-12-22

**Authors:** Marta Merli, Lorenzo Sardelli, Nicolò Baranzini, Annalisa Grimaldi, Emanuela Jacchetti, Manuela Teresa Raimondi, Francesco Briatico-Vangosa, Paola Petrini, Marta Tunesi

**Affiliations:** ^1^ Department of Chemistry, Materials and Chemical Engineering “G. Natta”, Politecnico di Milano, Milan, Italy; ^2^ Department of Biotechnology and Life Sciences, University of Insubria, Varese, Italy

**Keywords:** 3D-printing, reactive printing, 3D-bioprinting, internal gelation, rheology, printability optimization, collagen, cell viability

## Abstract

**Introduction:** In the view of 3D-bioprinting with cell models representative of neural cells, we produced inks to mimic the basic viscoelastic properties of brain tissue. Moving from the concept that rheology provides useful information to predict ink printability, this study improves and expands the potential of the previously published 3D-reactive printing approach by introducing pH as a key parameter to be controlled, together with printing time.

**Methods:** The viscoelastic properties, printability, and microstructure of pectin gels crosslinked with CaCO_3_ were investigated and their composition was optimized (i.e., by including cell culture medium, HEPES buffer, and collagen). Different cell models representative of the major brain cell populations (i.e., neurons, astrocytes, microglial cells, and oligodendrocytes) were considered.

**Results and Discussion:** The outcomes of this study propose a highly controllable method to optimize the printability of internally crosslinked polysaccharides, without the need for additives or post-printing treatments. By introducing pH as a further parameter to be controlled, it is possible to have multiple (pH-dependent) crosslinking kinetics, without varying hydrogel composition. In addition, the results indicate that not only cells survive and proliferate following 3D-bioprinting, but they can also interact and reorganize hydrogel microstructure. Taken together, the results suggest that pectin-based hydrogels could be successfully applied for neural cell culture.

## 1 Introduction

Rendering selected aspects of the *in vivo* complexity in a representative laboratory model can greatly enhance our understanding of physiological and pathological phenomena and provide critical and cost-effective tools for diagnostic or high throughput screening, drug discovery and the development of medical devices (Peneda Pacheco et al., 2021). However, this is particularly challenging when modelling nervous tissue. The variety and plurality of topographical and biochemical stimuli driving its development and functions have fascinated the scientific community (Papadimitriou et al., 2020). However, this poses several questions on how it is possible to model nervous tissue.

In the route to the setting up of reliable and accurate *in vitro* engineered models of neural tissue, hydrogel-based 3D models have produced concrete advancements towards this end (Antill-O’Brien et al., 2019). Indeed, although 2D models are inexpensive and highly reproducible, they lack sufficient complexity to gain insight into the biological phenomena driving brain functionality (Hopkins et al., 2015). In contrast, hydrogels provide a rudimentary 3D extra-cellular matrix to reproduce more physiological-*like* conditions. Both natural (e.g. alginate, chitosan, collagen) and synthetic (e.g. polyethylene glycol, polycaprolactone) polymers have been exploited (Oliveira et al., 2019), eventually coupled with glial scar-inhibiting (Lee et al., 2010) or neurotrophic factors (Taylor et al., 2006). More recently, brain decellularized extracellular matrix was also proposed to match the physical (e.g. stiffness) and biochemical properties of native tissue, and modulate biological functions (Bae et al., 2021; Zhang et al., 2021). As an alternative, blending is a common approach to design composite materials with improved properties (Zhao et al., 2020), with one polymer providing suitable mechanical features, while the other(s) promoting cell adhesion and viability.

However, a third dimension is not enough to model highly hierarchical tissues such as the brain. Proper control of the architecture, pore interconnectivity and viscoelastic properties of hydrogels, as well as the distribution of different cell populations in the 3D environment, is still an open challenge to increase the reliability of *in vitro* models of the brain (Samanipour et al., 2022). For the fabrication of highly reproducible layered-based multicellular architectures with positional control over biomaterials and cells (Qiu et al., 2020), 3D-bioprinting technologies have emerged. As the products can be shaped on the architecture of a defect, bioprinting holds a great potential for localized pathological conditions, like stroke or traumatic brain injury. Especially in a delicate environment, such as the central nervous system, direct printing on the patient could improve the integration of engineered tissue with surrounding tissue (Mehrotra et al., 2019), pushing forwards personalized healthcare (Vaz and Kumar, 2021).

Due to the similarity with the sugar-based macromolecules in the extracellular matrix of native tissues, polysaccharide-based inks are extremely common in 3D-bioprinting. Engineering their formulation and properties is fundamental to promote cell viability and easily pattern the constructs (Paxton et al., 2017). To reach the first goal, several polysaccharides require grafting with peptides such as arginine-glycine-aspartic acid (RGD) or tyrosine-isoleucine-glycine-serine-arginine (YIGSR) moieties; for the second goal, the control of crosslinking offers the possibility to tailor and shape the constructs. For example, Schwann cells were loaded in a peptide-conjugated alginate solution then crosslinked by ionotropic external gelation with calcium ions produced by the dissolution of CaCl_2_, and the system sustained cell viability (Sarker et al., 2019). As previously reported, RGD-functionalization of gellan gum proved to be effective to bioprint cortical neurons in layered structures (Lozano et al., 2015). In this case, the bioink/cell suspension and the crosslinker (e.g. CaCl_2_ or 5× Dulbecco’s modified Eagle’s medium, DMEM) were loaded into different syringes and flowed through silicone tubing before automatic mixing and extrusion. According to the hypothesis that weakly crosslinked bioinks before printing can shield from potential damage to cell membranes, Lindsay et al. (2019) also pursued this strategy, but they added a post-printing crosslinking step to stabilize the constructs. They printed neural progenitor cells into RGD-functionalized alginate that had been pre-crosslinked with CaSO_4_, and then covered the samples with culture medium supplemented with CaCl_2_ for a further crosslinking step. Overall, these crosslinking approaches rely on the diffusion of highly soluble calcium ions and produce inhomogeneous hydrogels (Skjåk-Bræk et al., 1989; Secchi et al., 2014). Indeed, polymer concentration decreases from the interface with the crosslinking solution to the center of the gels (Secchi et al., 2014), with impact on both cell distribution along the fiber sections and gas/nutrient diffusion.

Alginate is the most widely applied polysaccharide for cell delivery. However, it has limited chemical stability in culture media, where calcium chelators (e.g. phosphate, lactate, citrate) and monovalent cations (e.g. sodium and magnesium) may displace calcium ions. In contrast, Ca-pectinate gels are less sensitive to chemical agents and represent a better choice for cell embedding (Wan-Ping et al., 2011). Pectin, which is a versatile class of anionic and branched polysaccharides found in the cell walls of land-based plants, is regarded as the most structurally and functionally complex polysaccharide in nature (Liang and Luo, 2020). Due to its relatively low cost, stability, and gelation properties, pectin is traditionally used in food industry (Vancauwenberghe et al., 2018). Its bioavailability and biological features have also favored its use in the pharmaceutical industry (e.g. drug administration, Lara-Espinoza et al., 2018; films with antimicrobial properties, Kumar et al., 2020; wound dressing, Andriotis et al., 2020) and in tissue engineering/regenerative medicine (Munarin et al., 2011; Pereira et al., 2018; Campiglio et al., 2021).

Although pectin exhibits several bioactive properties that could be favorably applied in neural tissue engineering, its exploitation in this field is lacking. It displays metal-binding ability (Lessa et al., 2020), cancer inhibition due to the close interaction with galectin-3 (Gao et al., 2013), and mucoadhesiveness (Sriamornsak et al., 2010). Heavy metals are involved in the control of oxidative stress (Giacoppo et al., 2014; Giampietro et al., 2018), a mechanism that leads to neurodegeneration (Bedini et al., 2021). Galectin-3 is expressed by reactive microglia. It has emerged as a potential biomarker for Alzheimer’s and Parkinson’s diseases, but it also promotes inflammation in traumatic brain injury (García-Revilla et al., 2022). Finally, mucoadhesiveness could be exploited for the intranasal delivery of cells to the brain (Danielyan et al., 2009). Furthermore, pectin exhibits conductive properties (Dennis et al., 2022) that could represent an effective starting point for enhancing neuronal cell adhesion and neurite formation (Zhong and Bellamkonda, 2008), although its exploitation for neural cell culture is still lacking.

Like gellan gum and alginate, low methoxyl pectin shows cation-binding capacity. It interacts with divalent cations *via* its non-methyl-esterified galacturonic acid units (Celus et al., 2018) to form ionic-bound gels stabilized by non-covalent crosslinks (external gelation). However, another mechanism is possible (i.e. internal gelation). It relies on the slow, progressive dissolution of poorly soluble calcium salts, such as CaCO_3_ or calcium phosphate particles (Munarin et al., 2014), which are homogeneously mixed with pectin solution. The distinctive features of internal gelation are the time-dependent variation of viscoelastic properties as well as the production of homogeneous networks, which are more advantageous for cell culture. A key factor to drive the crosslinking kinetics is pH (Burey et al., 2008; Moreira et al., 2014), because acidic pH drives the dissolution of calcium salts. Nevertheless, as with external gelation, also in the case of internal crosslinking, polysaccharides are described to be combined with other polymer components or subjected to post-printing treatments such as crosslinking by UV light to achieve suitable viscoelastic properties and stability (Li et al., 2020; Kim et al., 2021).

In this study, we propose a highly controllable method to optimize the printability of internally crosslinked polysaccharides, without the need for additives or post-printing treatments. Starting from the concept that rheology provides useful information to predict ink printability, we expanded the 3D-reactive printing approach (Sardelli et al., 2021) by introducing pH as a key parameter to be controlled, together with printing time. We investigated the suitability of the proposed inks for 3D-printing *a priori*, by analyzing the viscoelastic properties of pectin gels crosslinked with CaCO_3_. Ink composition was optimized in the view of applications with or without cell models representative of the major brain cell populations, for example by including cell culture medium and collagen. Then, 3D-bioprinted cell-laden constructs were produced according to the optimized printing conditions. Finally, the possibility of exploiting the selected ink for 3D-printed brain models was studied by evaluating cell viability over time.

## 2 Materials and methods

### 2.1 Materials

Low methoxyl pectin from citrus fruits (classic CU 701, batch 01907714) was kindly gifted by Herbstreith & Fox (Neuenbűrg, Germany) and stored at −20°C. Sodium bicarbonate was purchased from Zeta Farmaceutici (Sandrigo, Italy), calcium carbonate (CaCO_3_, code 2117, batch 180,575) from Caesar & Loretz GmbH (Hilden, Germany) and 0.9% w/v sodium chloride (NaCl) from Eurospital (Trieste, Italy). N-2-hydroxyethylpiperazine-N′-2-ethanesulfonic acid (HEPES) solution (pH 7.0–7.6), collagen solution from bovine skin (3 mg/ml, batch SLCH0781), phosphate buffered saline (PBS), resazurin sodium salt, sodium citrate tribasic, sodium hydroxide and reagents for microstructural characterization were obtained from Sigma-Aldrich (Merck KGaA, Darmstadt, Germany). Plasticware was purchased from Corning (Corning, NY, United States), while 3-(4,5-dimethylthiazol-2-yl)-5-(3-carboxymethoxyphenyl)-2-(4-sulfophenyl)-2H-tetrazolium (MTS) was Promega (Madison, WI, United States). Reagents for cell culture and confocal microscopy were obtained from Thermo Fisher Scientific (Waltham, MA, United States).

### 2.2 Experimental procedures

#### 2.2.1 Material preparation

For the sake of clarity, inks were labelled as *PxCayColl-Z*, where *x* represents pectin concentration (% w/v), *y* is CaCO_3_ concentration (mmol), Coll highlights the possible presence of collagen, and *Z* is the solvent for pectin solutions and CaCO_3_ suspensions (Table 1). For P_2.4_Ca_20_-NaCl and P_2.4_Ca_35_-NaCl, 2.4% w/v pectin was dissolved overnight in 0.9% w/v NaCl. To partially neutralize the carboxyl groups of pectin backbone, while preventing β-elimination, 20 mM NaHCO_3_ was progressively added. pH was adjusted to 3.4 ± 0.1 (pHmeter Edge^®^, Hanna Instruments, Woonsocket, RI, United States) with 0.75 M NaHCO_3_. To promote hydrogel formation, CaCO_3_ suspensions (20 or 35 mM) were prepared in 0.9% w/v NaCl and mixed with pectin (1:1.5, volumetric ratio).

**TABLE 1:** Hydrogel labeling. Hydrogels were labelled as *PxCayColl-Z*, where *x* represents pectin concentration, *y* is CaCO_3_ concentration, Coll highlights the possible presence of collagen, and *Z* is the solvent for pectin solutions and CaCO_3_ suspensions.

Hydrogels (*PxCayColl-Z*)	*x,* pectin (% w/v)	*y,* CaCO_3_ (mmol)	*Coll,* COLLAGEN	*Z,* medium for pectin and CaCO_3_
P_2.4_Ca_20_-NaCl	2.4	20	Not present	0.9% w/v NaCl
P_2.4_Ca_35_-NaCl		35		
P_4_Ca_20_-DMEM	4	20		DMEM
P_3.8_Ca_20_-DMEM	3.8			
P_3.8_Ca_20_Coll-DMEM HEPES			Present	DMEM supplemented with HEPES

For P_3.8_Ca_20_-DMEM and P_4_Ca_20_-DMEM, pectin (3.8% or 4% w/v, respectively) was dissolved in high glucose DMEM (code 10938-025) supplemented with 10% v/v fetal bovine serum (FBS), 2 mM l-glutamine, 100 U/ml penicillin and 100 μg/ml streptomycin sulfate. pH was adjusted to 3.5 ± 0.1. CaCO_3_ suspensions (20 mM) were prepared in the same medium and mixed with pectin (1:1.5, volumetric ratio).

For P_3.8_Ca_20_Coll-DMEM HEPES, 3.8% w/v pectin was dissolved in DMEM also supplemented with 10 mM HEPES. pH was adjusted to 3.65 ± 0.1. CaCO_3_ suspensions (20 mM) were prepared in the same medium, mixed with pectin (1:1.5, volumetric ratio) and 3 min later with collagen 2.16 mg/ml (1:0.25, volumetric ratio). Collagen solution was obtained by diluting eight parts v/v bovine collagen with one part v/v PBS 10x and one part v/v 0.1 N NaOH, and then by mixing with DMEM HEPES (9:1, volumetric ratio).

For cell experiments, pectin was disinfected by washing it for three times (15 min/each) in ethanol and drying it in a laminar flow cabinet, while CaCO_3_ was heated overnight in an oven at 121°C.

#### 2.2.2 Rheological characterization and pH measurements

Rheological characterization was performed with a rotational rheometer (Modular Compact Rheometer MCR 502, Anton Paar, Graz, Austria) equipped with parallel-plate geometry (diameter: 25 mm; working gap: 0.5 mm). Experiments were run at 25 ± 0.01°C, controlling the temperature with a Peltier system.

To assess their reproducibility, before mixing with CaCO_3_, the viscosity of pectin solutions was measured in steady state shear experiments, which were performed at shear rates increasing from 0.1 to 100 s^−1^. Gelation kinetics was investigated by oscillatory time sweeps at 1.0 Hz and 0.5% shear strain amplitude for 140 min from the instant (t = 0) in which pectin was mixed with CaCO_3_. To ensure measurements in the linear regime, the linear viscoelastic region (LVR) was defined preliminarily to other tests by applying oscillatory shear amplitude ramps (logarithmic increase from 0.01 to 1000%, frequency 1.0 Hz) to fully gelled samples (i.e. 24 h after mixing pectin with CaCO_3_). The limit of the LVR was defined as the maximum shear strain amplitude after which the storage (*Gʹ*) and loss (*Gʺ*) moduli start changing from the previous constant value. During the first hour of crosslinking, pH was assessed every 5 min.

To study the time dependence of flowability and structure recovery within a time window suitable for 3D-bioprinting, samples were extruded in a Petri dish immediately after mixing (0 min), moved to the rheometer with a spatula and tested after 0, 30, 60 min crosslinking. Flowability was deduced from the viscosity curve obtained in a steady state shear test at shear rates increasing from 0.01 to 2,000 s^−1^. Only data acquired from the lowest shear rate to the maximum shear rate not inducing material removal from the rheometer geometry, was considered.

Hydrogel ability to recover their state after injection was estimated by the following three-step oscillation protocol: first, an oscillatory test was carried out for 100 s at 1.0 Hz and 0.5% strain amplitude to assess the pristine dynamic mechanical properties (*Gʹ*, *Gʺ*). Second, a 100% amplitude strain was applied at the same frequency for 100 s to cause a possible structural breakdown, and finally a third oscillatory step was carried out for 200 s in the same conditions as the first one to measure the recovery of *Gʹ*, *Gʺ* and thus assess the material ability to recover its pristine behavior and relevant microstructure.

The stress required to extrude the material was estimated from the measurements of the yield stress after 60 min crosslinking. Oscillatory tests were run at 1.0 Hz by increasing stress amplitude from 0.1 to 100 Pa. The yield stress was defined as the value of the shear stress at which *Gʹ* = *Gʺ* and the hydrogels undergo a transition from a solid- (*Gʹ* > *Gʺ*, tanδ < 1) to a liquid-*like* behavior (*Gʹ* < *Gʺ*, tanδ >1).

#### 2.2.3 Printability evaluation

Inks (2.5 ml in 3 ml syringes) were printed with the pneumatic-based extrusion bioprinter Inkredible^+™^ (Cellink, Gothenburg, Sweden) using conical 32 mm-length nozzles. The process applied a predefined code in Repetier-Host (Hot-World GmbH & Co. KG, Willich, Germany) operating in Slic3r (https://slic3r.org). Before cell loading or printing, inks were centrifuged (e.g., 800 rpm for 5 min) to remove air bubbles. Infill pattern, printing speed and layer height were varied to print fibers, two-layer geometries and five-layer grids.

**FIGURE 1:**
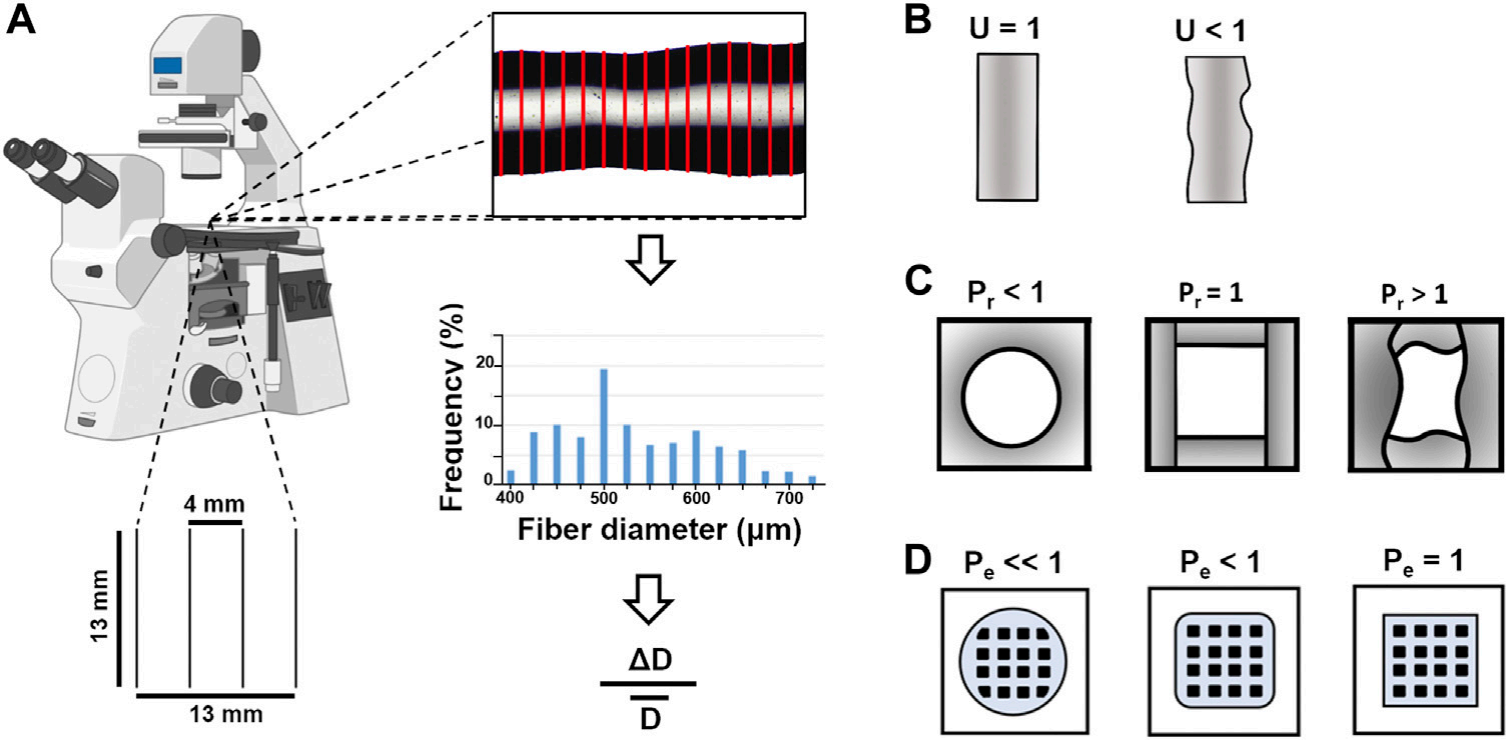
Representative sketches of the **(A)** method applied to measure the diameter of the single fibers printed at 0, 30, and 60 min in a 13 × 13 mm grid (10% infill). ΔD is the standard deviation of computed diameters, $\bar{D}$ is the mean diameter **(B)** geometry of fibers with uniformity factor *U* equals to or lower than 1. The lower *U* is, the more inhomogeneous the fiber cross-section is **(C)** geometry of pores with pore factor (*P*_*r*_, Sardelli et al., 2021) lower, equals or greater than 1. *P*_*r*_ < 1 suggests roundish pores, *P*_*r*_ = 1 identifies square pores and describes the ideal situation, while *P*_*r*_ > 1 refers to irregularly-shaped pores **(D)** geometry of two-layer grids with perimeter coefficient (*P*_*e*_, Sardelli et al., 2021) lower than or equal to 1. *P*_*e*_ = 1 describes the ideal situation.

Single fibers were printed after 0, 30, 60 min crosslinking (Figure 1A). Inks in 0.9% w/v NaCl were printed with 410 or 250 μm nozzles (22 or 25 G, respectively) at 10, 15, 25 mm/s; inks in DMEM w/or w/o HEPES were printed with 250 μm nozzles at 10, 15 mm/s. To study the effect of printing pressure on fiber diameter, pressure was progressively increased by 4 kPa during the same printing, starting from the minimal pressure for a continuous flow. We defined the maximum pressure as the highest pressure at which we could print without issues, such as extruding a considerable amount of material from the cartridge or blocking the printer. Immediately after printing and before shrinkage due to solvent evaporation, fibers were imaged by an optical microscope (Eclipse Ti2, Nikon, Tokyo, Japan). To account for diameter inhomogeneity during extrusion, the initial, middle and final parts of the fibers were dimensioned. For each image, fiber diameter was measured every 10 pixels by a custom plug-in of ImageJ (http://imagej.nih.gov/ij/) and 290 measurements were obtained to estimate diameter distribution and mean values. Fiber uniformity was evaluated by uniformity factor (*U*, Figure 1B), according to Eq. 1:

$$U=1-\frac{\Delta D}{\bar{D}}$$ (1)

where ΔD is the standard deviation (SD) of computed diameters and $\bar{D}$ is the mean diameter.

After 0, 30 min crosslinking, two-layer geometries (25 mm × 25 mm × 0.6 mm, 25% infill) were printed at 10, 15 mm/s with 250 μm nozzles. They were made up of two parallel layers superimposed perpendicularly to each other. Each layer was limited by a square border. While printing, pressure was manually adjusted to ensure uniformity and shape integrity. Constructs were imaged following printing. To assess shape fidelity, i.e. the shape retention of the printed construct as a whole compared to the original computer design (Gillispie et al., 2020), pore factor (*P*_*r*_, Figure 1C) and perimeter coefficient (*P*_*e*_, Figure 1D) were calculated according to Eqs. 2 and 3 (Sardelli et al., 2021):

$$P_{r}=\frac{{\left(Pore\;Perimeter\right)}^{2}}{16\cdot \left(Pore\;Area\right)}$$ (2)

$$P_{e}=\frac{1}{\left\{\left[\frac{1}{2}\cdot \left(\frac{\bar{L_{x}}}{L_{x}}\cdot \frac{\bar{L_{y}}}{L_{y}}\right)-1\right]+1\right\}\cdot \left[1+\frac{1}{2}\cdot \left(\frac{\Delta L_{x}}{L_{x}}+\frac{\Delta L_{y}}{L_{y}}\right)\right]}$$ (3)

where *L*_*x*_ (*L*_*y*_) is the theoretical length along the horizontal (vertical) axis, $\bar{L_{x}}$ ( $\bar{L_{y}})$ is the respective mean value for the printed geometry, and $\Delta L_{x}$ ( $\Delta L_{y}$ ) is its SD. After selecting the edges of the pores, the perimeter (µm) and the area (µm^2^) to be used in Eq. 2 were measured by ImageJ.

In the view of cell experiments, after a crosslinking time from 0 to 30 min, five-layer grids (25 mm × 25 mm x 1.50 mm, 25% infill) were printed with P_3.8_Ca_20_Coll-DMEM HEPES at 10, 15 mm/s with 250 μm nozzles. After printing, grids were imaged and the results were compared to establish the optimal speed and crosslinking time for 3D-bioprinting.

#### 2.2.4 Cell culture

SH-SY5Y human neuroblastoma cells (ATCC^®^ code CRL-2266™), C8-D1A mouse astrocytes (ATCC^®^ code CRL-2541™), and HOG human oligodendroglioma cells (EMD Millipore, Merck, code SCC163) were grown in high-glucose DMEM (code 10938-025) supplemented with 10% v/v FBS, 2 mM l-glutamine, 100 U/ml penicillin and 100 μg/ml streptomycin sulfate. HMC3 human microglial cells (ATCC^®^ code CRL-3304™) were grown in Advanced minimum essential medium (code 12492-013) supplemented with 10% v/v FBS and 2 mM l-glutamine. All cell lines were cultured at 37°C, 5% CO_2_ in a humidified atmosphere. Medium was refreshed every two to 3 days and cells were split twice a week.

#### 2.2.5 Indirect cytocompatibility: MTS assay

After preparation, P_4_Ca_20_-DMEM and P_3.8_Ca_20_Coll-DMEM were incubated with DMEM (code 10,938–025) supplemented with 10% v/v FBS, 2 mM l-glutamine, 100 U/ml penicillin and 100 μg/ml streptomycin sulfate. After 1, 4, 24, 72 h and 7 days, supernatants were replaced with fresh medium. Cells (62.50 × 10^3^ C8-D1A, HMC3, HOG/cm^2^; 93.75 × 10^3^ SH-SY5Y/cm^2^) were plated in 96-well plates. The following day, cells were incubated with the supernatants, while controls were cultured in standard medium. After 24 h, cell viability was evaluated by MTS assay. Supernatants were replaced with medium supplemented with 10% v/v MTS. After 3 h incubation, the optical density was measured at 490 nm (reference wavelength 630 nm) by a spectrophotometric plate reader (Infinite 200 PRO, Tecan, Männedorf, Switzerland). The results were normalized to those of the controls.

#### 2.2.6 Microstructural characterization: Transmission electron microscopy

After mixing, samples (0.4 ml) were prepared into cylindrical molds (inner diameter: 11.05 mm) in 12-well plates. The procedure was repeated for cell-loaded constructs, obtained by mixing 2.5 × 10^6^ C8-D1A cells with collagen solution (9:1, volumetric ratio), and then with pectin/CaCO_3_. The moulds were removed after 1 h.

The 3D organization of P_3.8_Ca_20_-DMEM and P_3.8_Ca_20_Coll-DMEM HEPES was evaluated by transmission electron microscopy (TEM, Tunesi et al., 2019). Samples were fixed in 2% glutaraldehyde in 0.1 M cacodylate buffer (pH 7.4) for 2 h, washed for several times in the same buffer, and post fixed in 1% osmium tetroxide for 1 h. After standard ethanol dehydration, specimens were embedded in Epon-Araldite 812 mixture. Ultrathin sections (80 nm thick) were obtained with a Reichert Ultracut S ultratome (Leica, Wien, Austria), placed on copper grids (300 mesh) and stained with uranyl acetate and lead citrate.

For the immunogold assay, cell-loaded samples were fixed in 4% p-formaldehyde and 0.5% glutaraldehyde in PBS for 2 h, and then dehydrated in ethanol series for resin embedding. Ultrathin sections were obtained as above and collected on gold grids (300 mesh). After etching with 3% NaOH in ethanol (Causton, 1984), sections were incubated for 30 min in blocking solution containing 1% bovine serum albumin, 2% PBS and 0.1% Tween. They were incubated with the polyclonal primary antibody rabbit anti-COL1α1 (rabbit polyclonal, EMD Millipore) diluted 1:20 in blocking solution. After several washings in PBS, the primary antibody was visualized after immunostaining for 1 h with the secondary goat anti-rabbit IgG (H + L)-gold conjugate antibody (particle size: 10 nm. GE Healthcare, Amersham, UK) diluted 1:50 in blocking solution. In control experiments, the primary antibody was omitted, sections were treated with bovine serum albumin and incubated only with the secondary antibody. Sections were counterstained with uranyl acetate in water. Samples were observed with a Jeol 1010 EX electron microscope (Jeol, Tokyo, Japan) and data was recorded with a MORADA digital camera system (Olympus, Tokyo, Japan).

#### 2.2.7 Direct cytocompatibility after 3D-bioprinting

Cells (2.5 × 10^6^ C8-D1A, HMC3, HOG; 3·10^6^ SH-SY5Y) were mixed to collagen solution (9:1, volumetric ratio), and then with pectin/CaCO_3_. P_3.8_Ca_20_Coll-DMEM HEPES was loaded into a cartridge and kept in ice until assembling on the bioprinter. Five-layer grids were printed at 10 mm/s with 250 μm nozzles in 12-well plates after a crosslinking time from 10 to 20 min and covered with 0.75 ml medium.

After about 60 min crosslinking, cell viability following printing was assessed by a trypan blue exclusion assay. To view cells more easily, dissociation of the hydrogel network was promoted by incubation with sodium citrate (20 mM in DMEM (0.2% w/v), ∼200 μL). An aliquot of cell suspension was mixed with trypan blue dye and counted with a Neubauer chamber. The percentage of live cells was calculated according to Eq. (4):

$$live\;cells\;\left(\%\right)=\frac{\sum_{i=1}^{4}N{}^{\circ}\;live\;cells}{\sum_{i=1}^{4}N{}^{\circ}\;cells}\cdot 100$$ (4)

where 4 is the number of squares in which cells were counted and *N° cells* is the total number of cells counted.

To stain cell nuclei of live and dead C8-D1A cells after bioprinting, samples were incubated for 10 min in fresh medium supplemented with 1 μM Hoechst 33342, 0.5 μM Calcein AM and 0.2 μM ethidium homodimer-1 dyes (Thermofisher, Italy). Live fluorescence images were acquired by a confocal microscope (Ar1^+^, Nikon, Tokyo, Japan), equipped with an incubator chamber and four wavelength diode lasers (*λ*_excitation_ = 405/488/561/640 nm). Stained cells were imaged with a ×10 objective, with 0.45 NA, 4WD. The pinhole was set to one Airy Unit. 1024 × 1024 pixels images were acquired as z-stack images. Samples were imaged with a 10 µm step, resulting in an acquisition depth of approximatively 1.3000 µm.

To investigate the potential of P_3.8_Ca_20_Coll-DMEM HEPES for 3D-bioprinted models of brain tissue, cell constructs were printed in Transwell^®^ permeable supports in 6-multiwell plates. After 60 min crosslinking, they were covered with 2.5 ml culture medium. On 1, 4, 24 h, 3 and 7 days, cell viability was evaluated by a resazurin assay. Samples were maintained in cell culture medium supplemented with 10% v/v resazurin 0.2 mg/ml in PBS for 3.5 h, then 100 μL supernatants were moved to a 96-well plate and fluorescence was measured at 560 nm (reference wavelength 590 nm, manual gain: 60) by a spectrophotometric plate reader. The analyses were also performed on cell-free samples, whose fluorescence was subtracted from the cell-loaded ones.

#### 2.2.8 Statistical analysis

Results were reported as mean ± SD and analyzed with GraphPad Prism^®^, release 9 (GraphPad Software, La Jolla, CA, United States). The normality of data distribution was assessed by D’Agostino & Pearson test. For comparisons among groups, one-way analysis of variance (ANOVA) followed by Tukey’s multiple comparison test was performed. For comparisons between two groups, a two-tailed Mann-Whitney test was applied. Differences were considered as statistically significant when *p*-value <0.05 (*).

## 3 Results

In this study, we exploited the potential of a well-defined set of rheological analyses to study the viscoelastic properties of pectin solutions and gels. Our results predicted ink printability and expanded the 3D-reactive printing approach (Sardelli et al., 2021) by controlling both on printing time and pH. Both parameters were also fundamental to lay the grounds towards the development of pectin-based formulations as novel inks for 3D-bioprinting in neural tissue engineering-related applications.

### 3.1 Rheological characterization and pH measurements

**FIGURE 2:**
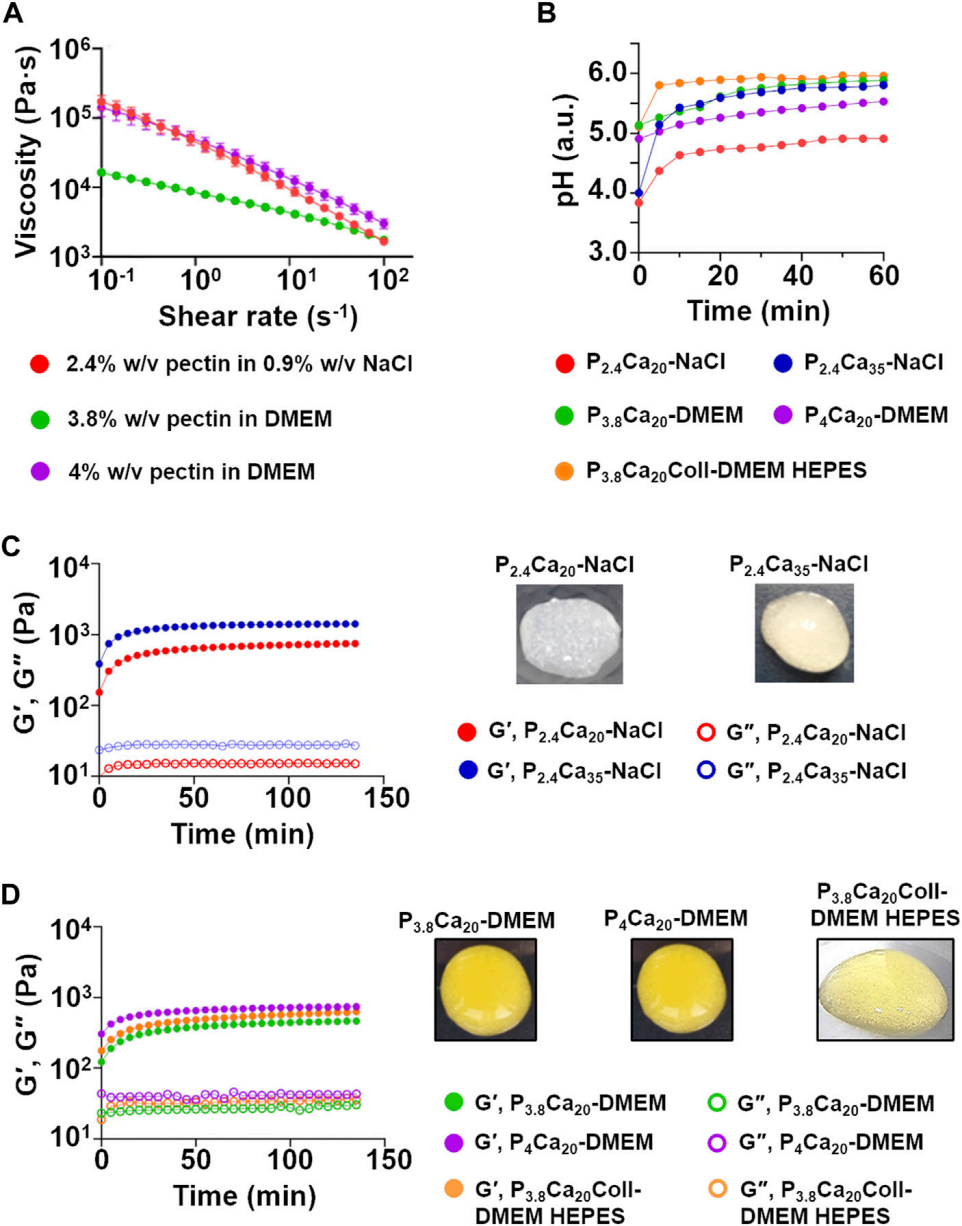
**(A)** Flow curves for 2.4% w/v pectin in 0.9% w/v NaCl, 3.8% w/v and 4% w/v pectin in DMEM. Mean ± SD, 12 replicates/condition **(B)** pH of the tested materials as a function of crosslinking time. Mean values, three replicates/condition **(C)** *G′, G″* as a function of crosslinking time for P_2.4_Ca_20_-NaCl and P_2.4_Ca_35_-NaCl. Mean values, three replicates/condition. Representative images of both materials at 1 h **(D)** *G′, G″* as a function of crosslinking time for P_3.8_Ca_20_-DMEM, P_4_Ca_20_-DMEM and P_3.8_Ca_20_Coll-DMEM HEPES. Mean values, three replicates/condition. Representative images of the gels at 1 h.

Pectin solutions exhibited a shear thinning behavior, independently of the solvent adopted (Figure 2A). In agreement with other studies (Kar and Arslan, 1999; Moreira et al., 2014), viscosity depended both on pectin concentration and pH. At all shear rates, viscosity was greater for 4% w/v than 3.8% w/v pectin in DMEM. At low shear rates, the flow curves for 2.4% w/v pectin in 0.9% w/v NaCl and 4% w/v pectin in DMEM were overlapping.

As previously suggested, pH regulation is fundamental to prevent metabolic alterations, maintain cell viability and exert signaling (Chesler, 2003; Flinck et al., 2018). However, the acidic pH of pectin solutions and hydrogels could limit their use for cell delivery (Moreira et al., 2014). For these reasons, pH was monitored over crosslinking time (Figure 2B). At all the time points, the higher amount of CaCO_3_ (35 mM) led to a faster increase of pH, producing hydrogels with a stiffer consistency (Figure 2C), but with residual deposits of calcium salts (e.g. for P_2.4_Ca_20_-NaCl, pH = 4.37 at 5 min and pH = 4.91 at 1 h; while 35 mM CaCO_3_ induced pH = 5.14 at 5 min and pH = 5.80 at 1 h). Dissolving pectin in DMEM (i.e. a buffered solution) was advantageous only in the first time point (at 5 min, for P_2.4_Ca_20_-NaCl pH = 4.37. In the presence of DMEM, pH = 5.26, Figure 2B). The addition of HEPES and Coll increased the pH both at shorter and longer time points (for P_3.8_Ca_20_Coll-DMEM HEPES, pH = 5.81 at 5 min and pH = 5.97 at 1 h). Although small, these differences are fundamental. When external pH is lowered from physiological values, cell membranes are deformed, processes stop moving or are retracted, cytoplasmic components start aggregating, and mitosis is paused. How long cells can withstand this condition depends on the acidity of the environment, but for pH values close to 6, slight differences (e.g. ∼ 0.3 pH units) can extend this time of some hours (Taylor, 1962).

The different composition and pH values affect the crosslinking kinetics, as was observable by time sweeps (Figures 2C, D). For all the materials, a solid-*like* state (*G′* > *G″*) was observed from t = 0, indicating that gelation occurred before starting the scans. P_2.4_Ca_35_-NaCl showed the highest viscoelastic properties (at the end of the scans, *G*´ = 1.41.10^3^ Pa; *G″* = 0.27.10^3^ Pa). *G′*, but for some materials also *G″*, increased over time, suggesting that crosslinking continued over time and gels progressively stiffened, in agreement with previous studies (Secchi et al., 2014). After a rapid rise in the first 15 min, the increase in stiffening slowed down and it was noticeable only over longer time frames. After 1 h, *G′* was equal to 80%–90% of its value at 140 min, when crosslinking could be considered as complete. In contrast, *G″* did not vary or only slightly varied over time. At 3.8% w/v pectin concentration, DMEM reduced the crosslinking rate and collagen did not affect it. Based on these results and to identify a relevant time window for extrusion-based bioprinting, we set 60 min crosslinking as the threshold value to study hydrogel properties. Indeed, since 3D-bioprinting experiments were not performed inside a cell culture incubator (i.e. it was not possible to control the temperature, humidity and carbon dioxide concentration to maintain an optimal environment for cell growth), cell viability could not be ensured (the latter can only be ensured if cells are kept out of an incubator for short time frames).

**FIGURE 3:**
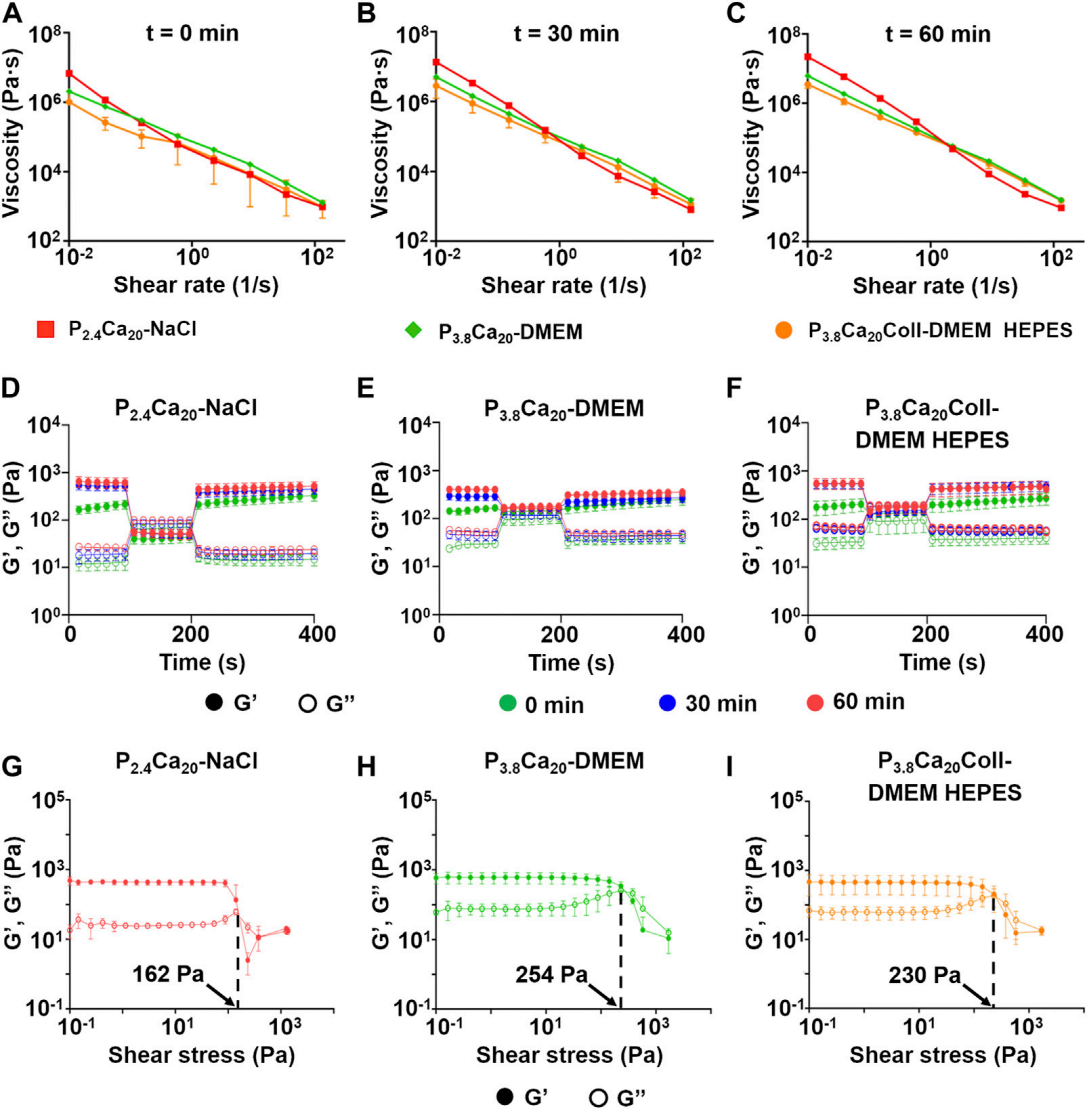
Prediction of ink extrudability from the flow curves at 0 **(A)**, 30 **(B)**, and 60 **(C)** min crosslinking for P_2.4_Ca_20_-NaCl, P_3.8_Ca_20_-DMEM, and P_3.8_Ca_20_Coll-DMEM HEPES. Mean ± SD, 4 replicates/condition; Recovery of *G′, G″* as a function of test time for P_2.4_Ca_20_-NaCl **(D)**, P_3.8_Ca_20_-DMEM **(E)**, and P_3.8_Ca_20_Coll-DMEM HEPES **(F)** after applying a 100% strain at 1 Hz for 100 s. Mean ± SD, 4 replicates/condition. *G′* and *G″* over shear stress after 60 min crosslinking for P_2.4_Ca_20_-NaCl **(G)**, P_3.8_Ca_20_-DMEM **(H)**, and P_3.8_Ca_20_Coll-DMEM HEPES **(I)**. The arrows indicate the yield stress. Mean ± SD, three replicates/condition.

Since extrusion-based printing requires the inks to flow through the nozzle/needle, their extrudability was studied (Figures 3A–C). As previously reported, the commonly accepted rheological indicator of extrudability is the viscosity, with higher viscosity leading to lower extrudability (Copus et al., 2022). Extrudability increased with the shear rate. In agreement with the rapid rise in dynamic moduli in the initial crosslinking phases, for a fixed shear rate extrudability at 0 min was greater than at 30 min, while the differences between 30 min and 60 min were negligible. At low shear rates, after 0 and 30 min crosslinking, P_3.8_Ca_20_Coll-DMEM HEPES showed the highest extrudability, while hydrogels in 0.9% w/v NaCl exhibited the lowest. At high shear rates, no difference was observed. To account for the influence of crosslinking kinetics, only the flow curves at 60 min were fitted with the power law model of Ostwald and de Waele (Eq. 5):

$$\eta =K{\left(\gamma ˙\right)}^{n-1}$$ (5)

where *η* is the viscosity, *K* the consistency index, γ˙ the shear rate and *n* the power law index. *n* ranges from 0 to 1, with one corresponding to a Newtonian (shear rate-independent) viscosity (Supplementary Table S1). As *K* rises, viscosity increases. Lower *K*, *n* values are related to a greater ease of extrusion and potentially to lower printing pressures (Liu et al., 2019). Although in disagreement with the printability results (see the following section), the lowest *K, n* values were calculated for P_2.4_Ca_35_-NaCl (*K*=6.50 × 10^4^, *n*=7.8 × 10^−3^). However, in support of the potential of this formulation for extrusion-based printing, the second lowest value of *K* was computed for P_3.8_Ca_20_Coll−DMEM HEPES (*K*=8.39 × 10^4^). Moreover, for gels in cell culture medium, *n*>0.1; while for the ones in 0.9% w/v NaCl, *n* < 0.01. We hypothesized that in the first situation (e.g. 20 mM CaCO_3_, DMEM and collagen) the power law was able to accurately models hydrogel behavior and to validate the shear thinning behavior, while in the second one (e.g., 35 mM CaCO_3_, 0.9% w/v NaCl) the extremely low value of *n*, if not related to a slippage between the rheometer plates, indicated a plug flow in the nozzle of the printer, rather than the flow of a liquid (Lopez Hernandez et al., 2021)^.^

After 0, 30, and 60 min crosslinking, *Gʹ* and *Gʺ* recovery was calculated (Figures 3D–F and Table 2) according to Eqs. 6 and 7, respectively; after applying a 100% strain for 100 s:

$$G^{\prime }\_Recovery\;\left(\%\right)=\left(\frac{G_{400}^{\prime }}{G_{100}^{\prime }}\right).100-100$$ (6)

$$G^{\prime \prime }\_Recovery\;\left(\%\right)=\left(\frac{G_{400}^{\prime \prime }}{G_{100}^{\prime \prime }}\right).100-100$$ (7)

where *Gʹ*_*400*_ (*Gʺ*_*400*_) is *Gʹ* (*Gʺ*) value at 400 s (i.e. at the end of the test) and *Gʹ*_*100*_ (*Gʺ*_*100*_) is *Gʹ* (*Gʺ*) value at 100 s (i.e. before applying a 100% strain for 100 s). To assess whether a deformation beyond the LVR affects hydrogel structure, at 150 s after beginning the test, tan(δ) was calculated according to Eq. 8:

Tanδ150=G150″G150′ (8)

where *Gʹ*_*150*_ (*Gʺ*_*150*_) is *Gʹ* (*Gʺ*) value at 150 s.

**TABLE 2:** *Gʹ_Recovery*, *Gʺ_Recovery*, and Tan(δ)_150_ for the proposed inks. They were calculated according to Eqs 6–8. The measurements were carried out at 0, 30, and 60 min crosslinking. Mean values, 4 replicates/condition.

Time (min)	Ink	Gʹ*_*Recovery	Gʺ*_*Recovery	Tan(δ)_150_
0	**P**_**2.4**_ **Ca**_**20**_ **-NaCl**	+52%	+14%	1.64
30		−14%	+2%	1.63
60		−15%	−6%	1.87
0	**P**_**2.4**_ **Ca**_**35**_ **-NaCl**	+22%	+19%	4.03
30		−15%	−11%	3.59
60		−12%	−2%	3.94
0	**P**_**4**_ **Ca**_**20**_ **-DMEM**	+44%	+29%	1.00
30		—	+1%	1.20
60		−5%	−2%	1.17
0	**P**_**3.8**_ **Ca**_**20**_ **-DMEM**	+48%	+29%	0.70
30		−6%	—	0.70
60		−12%	−5%	0.91
0	**P**_**3.8**_ **Ca**_**20**_ **Coll-DMEM HEPES**	+33%	+22%	0.69
30		−9%	−3%	1.25
60		−24%	−16%	1.09

When gels were subjected to an increasing shear immediately after preparation (t = 0), they not only recovered, but they even increased their viscoelastic properties (maximum values were about 50% for *Gʹ* and about 30% for *Gʺ*). The situation changed after 30 min crosslinking: except for P_4_Ca_20_-DMEM (no changes), *Gʹ* values did not recover after the deformation for the considered hydrogels. At this time point *Gʺ* values were constant or only slightly modified. After 60 min crosslinking, the inks did not recover their viscoelastic properties. P_3.8_Ca_20_Coll-DMEM HEPES showed the highest decrease in *Gʹ* (-24%) and *Gʺ* (-16%) values.

At all the crosslinking time points, tan(δ)_150_ was > 1 (liquid-*like* state) for inks in 0.9% w/v NaCl, while tan(δ)_150_ was <1 for DMEM samples w/o collagen, indicating a solid-*like* state. For the other materials, tan(δ)_150_ depended on crosslinking time. At 0 min, for P_4_Ca_20_-DMEM the deformation triggered a solid-to liquid-*like* transition (tan(δ)_150_ = 1), while P_3.8_Ca_20_Coll-DMEM HEPES maintained its solid-*like* behavior. At 30 min and 60 min, both the materials showed a liquid-*like* behavior. At 60 min, for P_3.8_Ca_20_Coll-DMEM HEPES, tan(δ)_150_ decreased and approached to 1 (1.09).

For shear stresses larger than the yield stress (Figures 3G–I), *G″* was higher than *Gʹ* and the sample started flowing. Therefore, the presence of a yield stress indicated that the hydrogel was capable to be extruded during printing.

Taken together, these results demonstrate that all the hydrogels efficiently recovered their viscoelastic properties when exposed to a strain beyond the LVR immediately after preparation, because in the early phases of crosslinking the general increase in dynamic moduli was fast. In agreement with the measured values of yield stress, the materials in cell culture medium required a higher deformation for a solid-to liquid-like transition. Between 30 and 60 min crosslinking, the rise in dynamic moduli during the recovery phase was considerably reduced and it could not compensate the effects of an extreme deformation. Except for P_3.8_Ca_20_-DMEM, all the formulations behaved as liquids, at the end of the recovery period. The exceptional behaviour of P_3.8_Ca_20_-DMEM could be explained by the fact that this formulation exhibited the greatest yield stress. The addition of collagen to P_3.8_Ca_20_-DMEM lowered the yield stress and improved the extrudability, with advantages for 3D-bioprinting.

### 3.2 Printability evaluation

**FIGURE 4:**
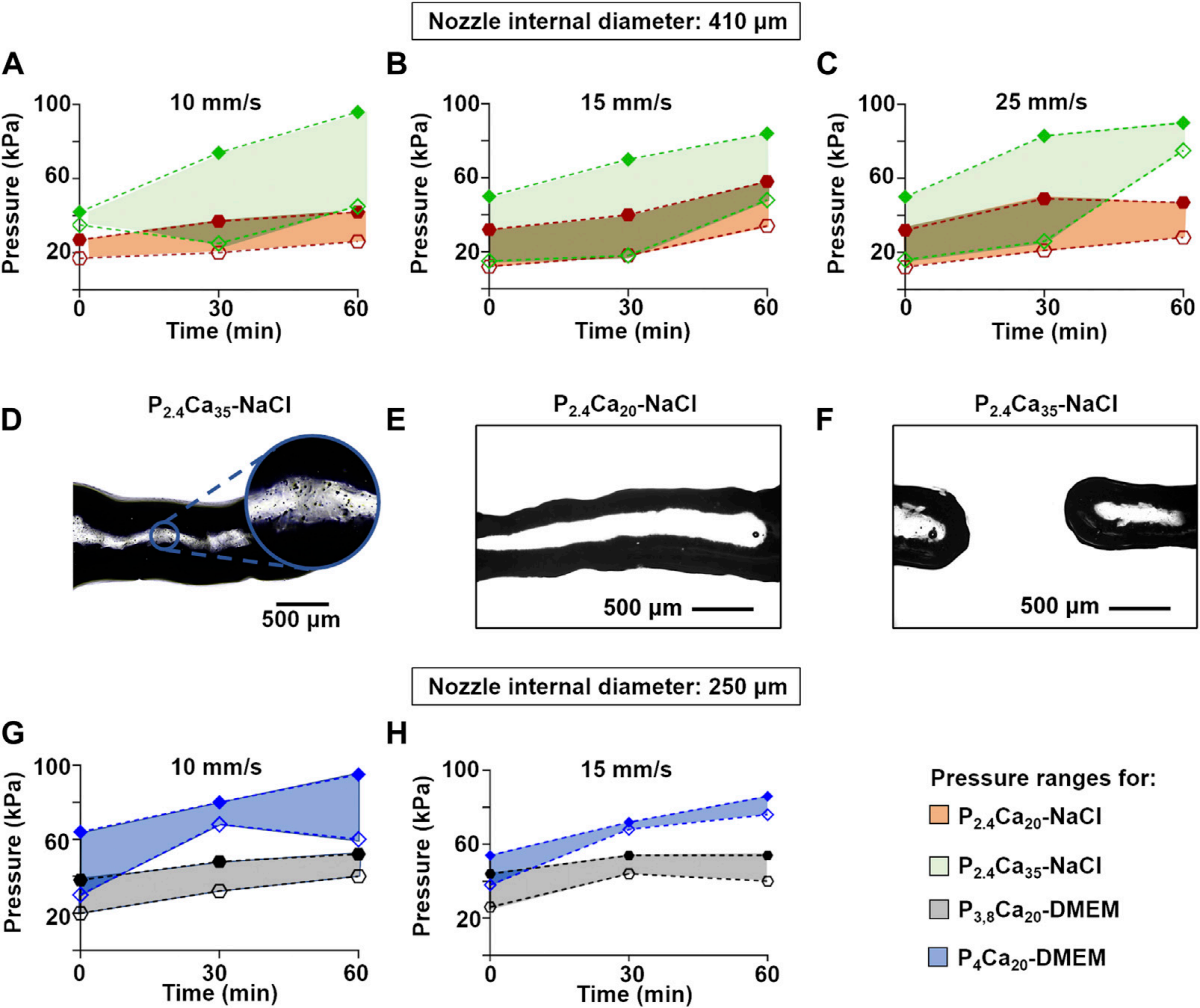
Minimum (empty indicators) and maximum (filled indicators) pressures as a function of crosslinking time to extrude single fibers of P_2.4_Ca_20_-NaCl and P_2.4_Ca_35_-NaCl with a 410 μm nozzle at 10 **(A)**, 15 **(B)**, and 25 **(C)** mm/s. The results were obtained after comparing 10 replicates/condition. The colored areas represent the printability windows; Representative optical images of **(D)** P_2.4_Ca_35_-NaCl printed fibers, showing calcium deposits **(E)** P_2.4_Ca_20_-NaCl printed fibers **(F)** P_2.4_Ca_35_-NaCl printed fibers, showing the lack of integrity. Scale bar = 500 μm; Minimum (empty indicators) and maximum (filled indicators) pressures as a function of crosslinking time to extrude single fibers of P_3.8_Ca_20_-DMEM and P_4_Ca_20_-DMEM with a 250 μm nozzle at 10 **(G)**, and 15 **(H)** mm/s. The results were obtained after comparing 10 replicates/condition. The colored areas represent the printability windows.

Since cell printing with high viscous inks (i.e. in hydrogels with high polymer content) requires high pressures and printing pressure can impair cell viability (Cidonio et al., 2019; Boularaoui et al., 2020), we studied the influence of speed and crosslinking on printing pressure (Figure 4).

The effect of CaCO_3_ content was investigated by printing a series of single fibers for P_2.4_Ca_20_-NaCl and P_2.4_Ca_35_-NaCl, i.e. for inks with the same pectin concentration (2.4% w/v) dissolved in the same solvent (0.9% w/v NaCl), but with different calcium content (20 or 35 mM)) (Figures 4A–C). At a fixed speed, both minimum and maximum pressures increased over crosslinking time. At a fixed crosslinking time, CaCO_3_ content affected maximum pressures. The higher amount of CaCO_3_ led to a greater gap between minimum and maximum pressures and required higher maximum pressures for extrusion. In contrast, comparable minimum pressures could be set at 0 min when printing at 15 or 25 mm/s, and at 30 min independently of speed. For a fixed CaCO_3_ content, pressures varied with speed. For P_2.4_Ca_35_-NaCl, fibers could be extruded at lower pressures when printing at 15 mm/s, while for P_2.4_Ca_20_-NaCl when printing at 10 or 15 mm/s. Since for both inks printing at 25 mm/s required pressures comparable or greater than printing at 10 or 15 mm/s, we excluded the fastest speed.

The microscopic observation of fibers with the higher calcium content (35 mM) highlighted dark spots (Figure 4D), indicating deposits of calcium carbonate. For both inks, fiber integrity decreased over crosslinking time and CaCO_3_ concentration (data not shown). Moreover, P_2.4_Ca_35_-NaCl frequently clogged the nozzle, requiring the interruption of the process (Figures 4E, F).

The effect of pectin concentration was investigated by printing a series of single fibers for P_4_Ca_20_-DMEM and P_3.8_Ca_20_-DMEM, i.e. for inks with the same calcium content (20 mM), but with different pectin contents (4% or 3.8% w/v) dissolved in the same solvent (DMEM) (Figures 4G, H). As for inks in 0.9% w/v NaCl, at a fixed speed, both minimum and maximum pressures increased over crosslinking time. Although there were only slight differences compared to inks in 0.9% w/v NaCl, at a fixed crosslinking time, pectin concentration affected both minimum and maximum pressures, with the extrusion of P_4_Ca_20_-DMEM requiring higher pressures than P_3.8_Ca_20_-DMEM. The printing speed also affected the results: for P_3.8_Ca_20_-DMEM, pressure values were lower when extruding at 10 mm/s than 15 mm/s; while for P_4_Ca_20_-DMEM lower values were set when printing at 15 mm/s.

**FIGURE 5:**
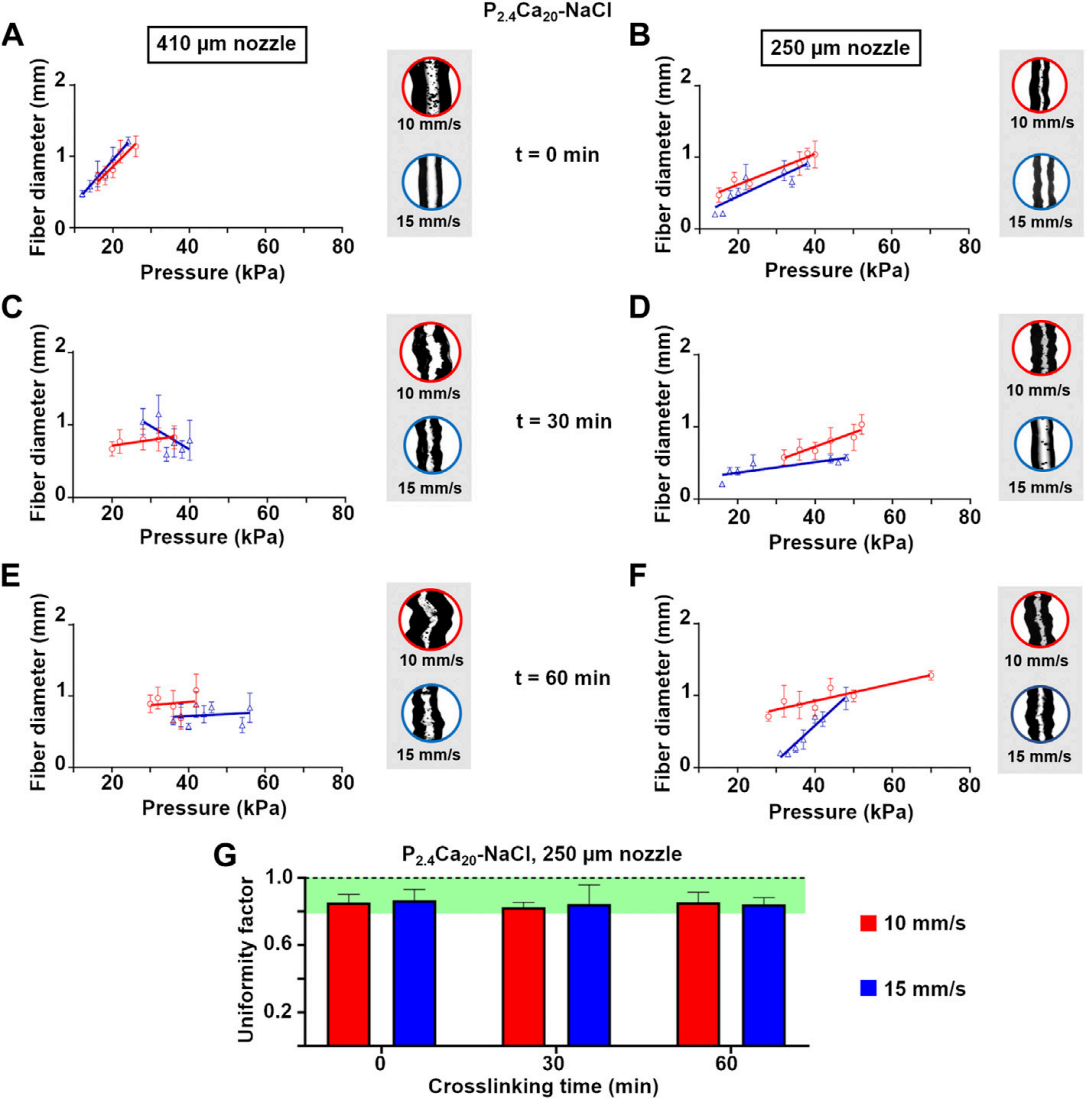
Fiber diameter as a function of printing pressure for P_2.4_Ca_20_-NaCl single fibers extruded with a 410 or 250 μm nozzle at 10 or 15 mm/s after 0 **(A and B)**, 30 **(C and D)**, and 60 **(E and F)** min crosslinking. Mean ± SD, six replicates/condition. For each condition, representative optical images of the central part of the fibers were reported **(G)** Uniformity factor (*U*) for P_2.4_Ca_20_-NaCl single fibers extruded with a 250 μm nozzle at 10 or 15 mm/s after 0, 30, and 60 min crosslinking. Mean ± SD, six replicates/condition.

The influence of pressure and speed on fiber diameter was related to the print resolution. At the beginning of crosslinking (0 min), the diameter of printed P_2.4_Ca_20_-NaCl fibers increased with pressure both at 10 or 15 mm/s (Figure 5A). After 30 min crosslinking (Figure 5C), pressure induced a change in diameter only at 10 mm/s. When crosslinking time reached 60 min, the shape was retained regardless of pressure (Figure 5E). Independently of speed, fibers were always larger than the nozzle for the minimum printing pressures (Supplementary Table S2). When the nozzle dimension was reduced from 410 μm to 250 µm (Figures 5B, D, F), fiber diameter (Supplementary Table S2) increased (or slightly increased) with increasing pressure, independently of crosslinking time. More specifically, the speed of 15 mm/s allowed to extrude P_2.4_Ca_20_-NaCl fibers thinner than the nozzle and with a basically constant diameter (∼ about 205 μm, mean value) from 0 to 60 min crosslinking.

Generally, printing through the smaller nozzle (250 µm) produced thinner fibers. Surprisingly, minimum pressures were generally comparable or even lower than the ones for extrusion through the larger nozzle. This result agrees with the shear thinning nature of P_2.4_Ca_20_-NaCl: a reduction in nozzle dimension, increases the shear strain, and decreases the viscosity, thus reducing printing pressure. For this reason, we selected 250 µm nozzles for further 3D-printing experiments.

The uniformity factor is an important parameter to compare the geometrical features of the set with the experimental results after printing. For P_2.4_Ca_20_-NaCl fibers extruded through 250 µm nozzles, the uniformity factor was always greater than 0.82 (Figure 5G). For all the crosslinking times, no differences (*p*-value>0.05) were found between 10 and 15 mm/s. The comparison of mean values showed greater uniformity factors for printing at 15 mm/s at both 0 and 30 min crosslinking. However, at this printing speed a lower number of fibers was available for the analysis. Indeed, when printing at 10 mm/s fiber integrity was greater than 90%; while it dropped to 50% in the tests at 15 mm/s (data not shown).

**FIGURE 6:**
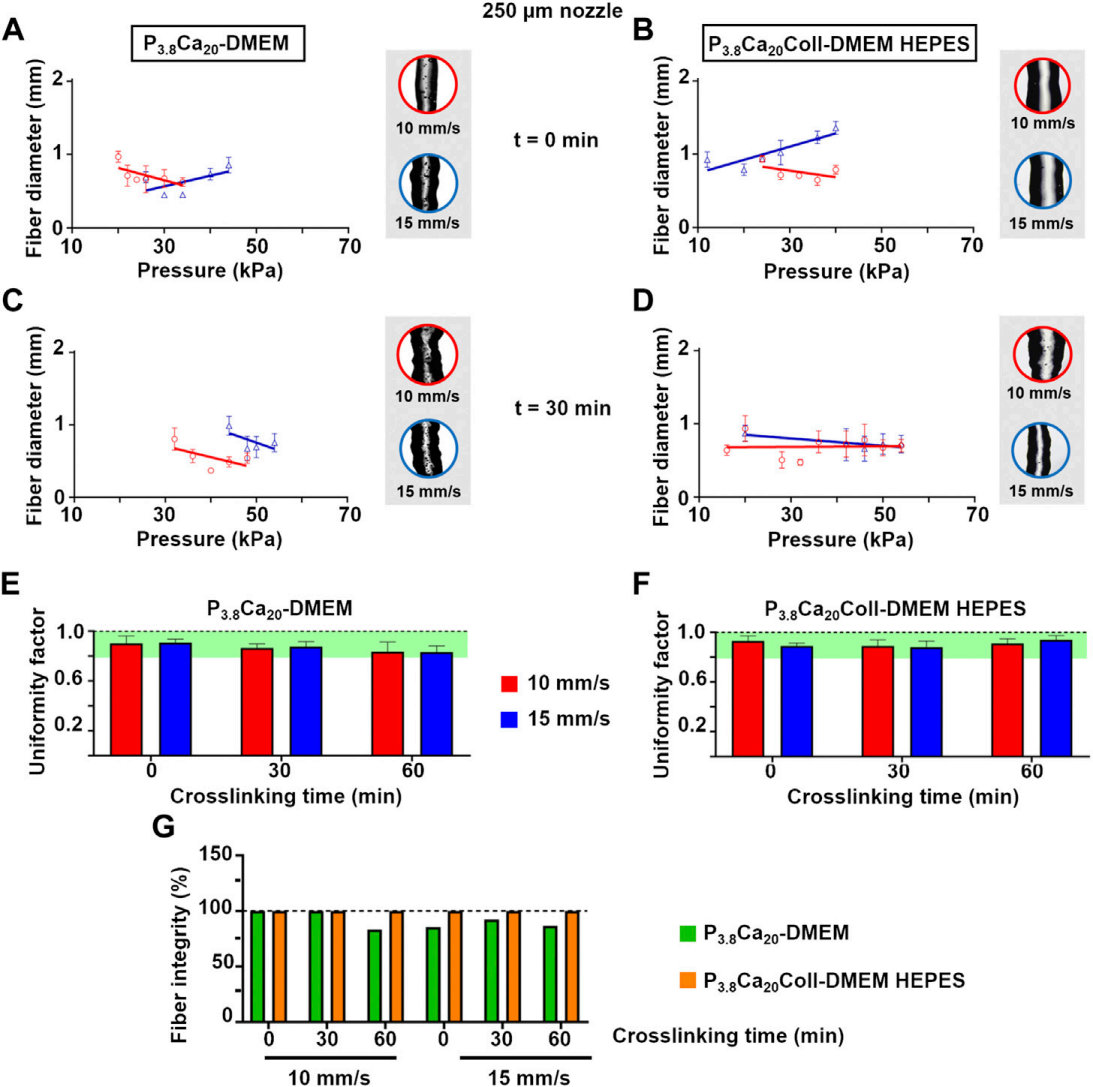
Fiber diameter as a function of printing pressure for P_3.8_Ca_20_-DMEM and P_3.8_Ca_20_Coll-DMEM HEPES single fibers extruded with a 250 μm nozzle at 10 or 15 mm/s after 0 **(A and B)**, 30 **(C and D)** min crosslinking. Mean ± SD, six replicates/condition. For each condition, representative optical images of the central part of the fibers were reported; Uniformity factor for: P_3.8_Ca_20_-DMEM **(E)** and P_3.8_Ca_20_Coll-DMEM HEPES single fibers **(F)** extruded with a 250 μm nozzle at 10 or 15 mm/s after 0, 30, and 60 min crosslinking. Mean ± SD, six replicates/condition **(G)** Fiber integrity for P_3.8_Ca_20_-DMEM and P_3.8_Ca_20_Coll-DMEM HEPES single fibers extruded with a 250 μm nozzle at 10 or 15 mm/s after 0, 30, and 60 min crosslinking. Mean ± SD, six replicates/condition.

For DMEM-based hydrogels either with or without collagen (i.e. P_3.8_Ca_20_-DMEM and P_3.8_Ca_20_Coll-DMEM HEPES, respectively), pressure and speed influenced fiber diameter (Figure 6, Supplementary Table S2). At 0 min (Figures 6A, B), the trends depended on speed, but not on the presence of collagen. For both inks, when printing at 10 mm/s, diameter decreased with increasing pressure, while the opposite was observed when printing at 15 mm/s. At 30 min, the trends were influenced by the presence of collagen. In the absence of collagen (i.e. for P_3.8_Ca_20_-DMEM), diameter decreased with increasing pressure, while in the presence of collagen (i.e. for P_3.8_Ca_20_Coll-DMEM HEPES), it was basically independent of pressure and speed (Figures 6C, D). At 60 min crosslinking, the trends varied with both speed and the presence of collagen. In the absence of collagen, when extruding at 10 mm/s, diameter increased with increasing pressure, while when printing at 15 mm/s, the opposite was observed (data not shown). Again, in the presence of collagen, the diameter of printed fibers was basically independent of pressure and speed (data not shown).

For crosslinking times compatible with cell viability outside the incubator during the printing process (i.e. 0 and 30 min), P_3.8_Ca_20_Coll-DMEM HEPES was extruded at lower pressures than P_3.8_Ca_20_-DMEM, thus representing an advantage for cell viability. The shift from 0.9% w/v NaCl to DMEM lowered print resolution, although the fibers were smoother and straighter. The addition of collagen to DMEM-based formulations improved fiber uniformity (Figures 6E, F), and integrity (Figure 6G). By extending the analysis up to 60 min, we were able to appreciate that collagen also improved recovery as crosslinking time increased. In fact, for P_3.8_Ca_20_-DMEM fibers extruded at both 10 mm/s and 15 mm/s, the uniformity factors decreased from 0.91 (0 min) to 0.87 (30 min) and 0.83 (60 min), while for P_3.8_Ca_20_Coll-DMEM HEPES fibers, they decreased from 0.91 (0 min) to 0.88 (30 min) and then increased to 0.92 (60 min).

**FIGURE 7:**
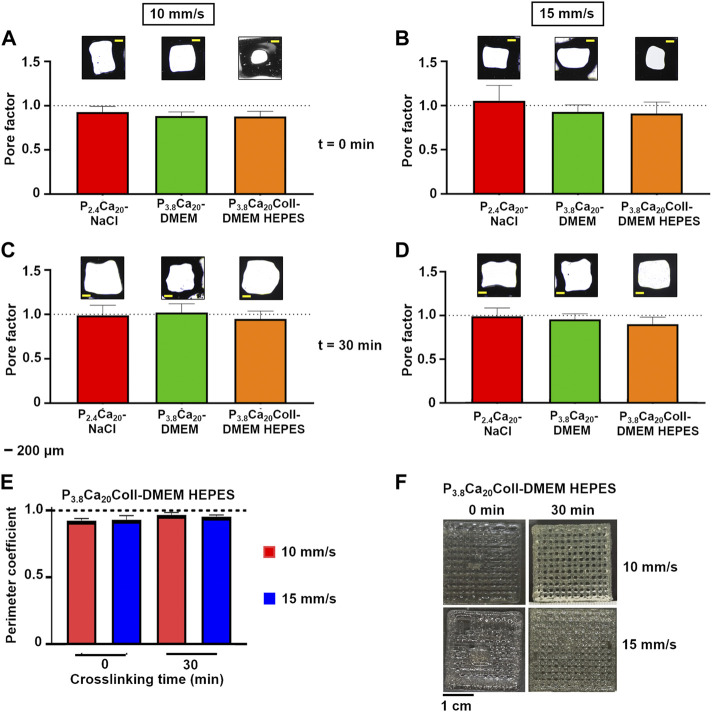
Pore factor for P_2.4_Ca_20_-NaCl, P_3.8_Ca_20_-DMEM and P_3.8_Ca_20_Coll-DMEM HEPES two-layer geometries printed after 0 and 30 min crosslinking at 10 **(A and B)** or 15 **(C and D)** mm/s. For each condition, representative optical images of the pores were reported. Scale bar = 200 μm **(E)** Perimeter coefficient for P_3.8_Ca_20_Coll-DMEM HEPES two-layer geometries printed after 0 and 30 min crosslinking at 10 or 15 mm/s **(F)** Representative images of five-layer grids printed after 0 and 30 min crosslinking at 10 or 15 mm/s. Scale bar = 1 cm.

As a proof of concept that it is possible to fabricate 3D-controlled shapes, we produced multilayered grids. As the uniformity factors did not allow for a unique selection of the printing speed, two-layer geometries were printed at different speeds and crosslinking times (Figures 7A–D). The minimum pressure values were set according to the ones for single fibers, but pressure could be increased during extrusion to allow for a continuous flow (Table 3). At 0 min, P_2.4_Ca_20_-NaCl grids were more uniform when printed at 10 mm/s than 15 mm/s (*p*-value<0.01); while at 30 min no differences were found with speed (*p*-value>0.05). More in general, the most uniform grids were printed at 10 mm/s after 30 min crosslinking (*P*_*r*_ = 0.99 ± 0.09). In these conditions, pores were the largest (i.e. fibers were the thinnest). For P_3.8_Ca_20_-DMEM, the results were reversed: at 0 min, no differences were found with speed (*p*-value>0.05); while at 30 min grids were more uniform when printed at 10 mm/s (*p*-value<0.05). Accordingly, for this ink the most uniform grids (*P*_*r*_ = 1.01 ± 0.14) were printed at 10 mm/s after 30 min crosslinking, and larger pores were obtained at the same crosslinking time when printing at 15 mm/s. However, when printing at 10 mm/s *P*_*r*_ increased with time (*p*-value<0.0001), whereas no differences were found with time at 15 mm/s (*p*-value>0.05). Even though for both crosslinking times no differences were found with speed (*p*-value>0.05), also for P_3.8_Ca_20_Coll-DMEM HEPES the most uniform grids were printed at 10 mm/s after 30 min crosslinking (*P*_*r*_ = 0.95 ± 0.09, mean ± SD). Again, an increase in the mean values of *P*_*r*_ (from 0.88 to 0.93) was observed over time at 10 mm/s. As regards *P*_*e*_ (Figure 7E), at both crosslinking times, no differences were found with speed (*p*-value>0.05). After 30 min crosslinking, it increased for both types of grids, independently of speed.

**TABLE 3:** Pressure ranges for printing P_2.4_Ca_20_-NaCl, P_3.8_Ca_20_-DMEM, and P_3.8_Ca_20_Coll-DMEM HEPES two-layered geometries with a 250 μm nozzle. Mean values, six replicates/condition.

Ink	Speed (mm/s)	Time (min)	Minimum pressure (kPa)	Maximum pressure (kPa)
**P**_**2.4**_ **Ca**_**20**_ **-NaCl**	10	0	15	40
		30	32	68
	15	0	18	30
		30	22	57
**P**_**3.8**_ **Ca**_**20**_ **-DMEM**	10	0	22	31
		30	32	62
	15	0	20	35
		30	44	74
**P**_**3.8**_ **Ca**_**20**_ **Coll-DMEM HEPES**	10	0	20	27
		30	27	42
	15	0	9	32
		30	17	41

Finally, we printed P_3.8_Ca_20_Coll-DMEM HEPES five-layer-grids (Figure 7F). Unlike the two-layer geometries, the speeds were not equivalent. For both crosslinking times, more uniform fibers and open porosities were obtained at 10 mm/s, but the shape fidelity increased over time.

Based on the results observed, we selected a crosslinking time between 10 and 20 min as the ideal printing time, and 10 mm/s as the ideal printing speed. They represented a compromise between the values of pore factor (*P*_*r*_, related to shape fidelity) and printing pressures (influencing cell viability, see the following section). Each five-layer-grid required about 100 s to be completed.

### 3.3 Indirect cytocompatibility: MTS assay

**FIGURE 8:**
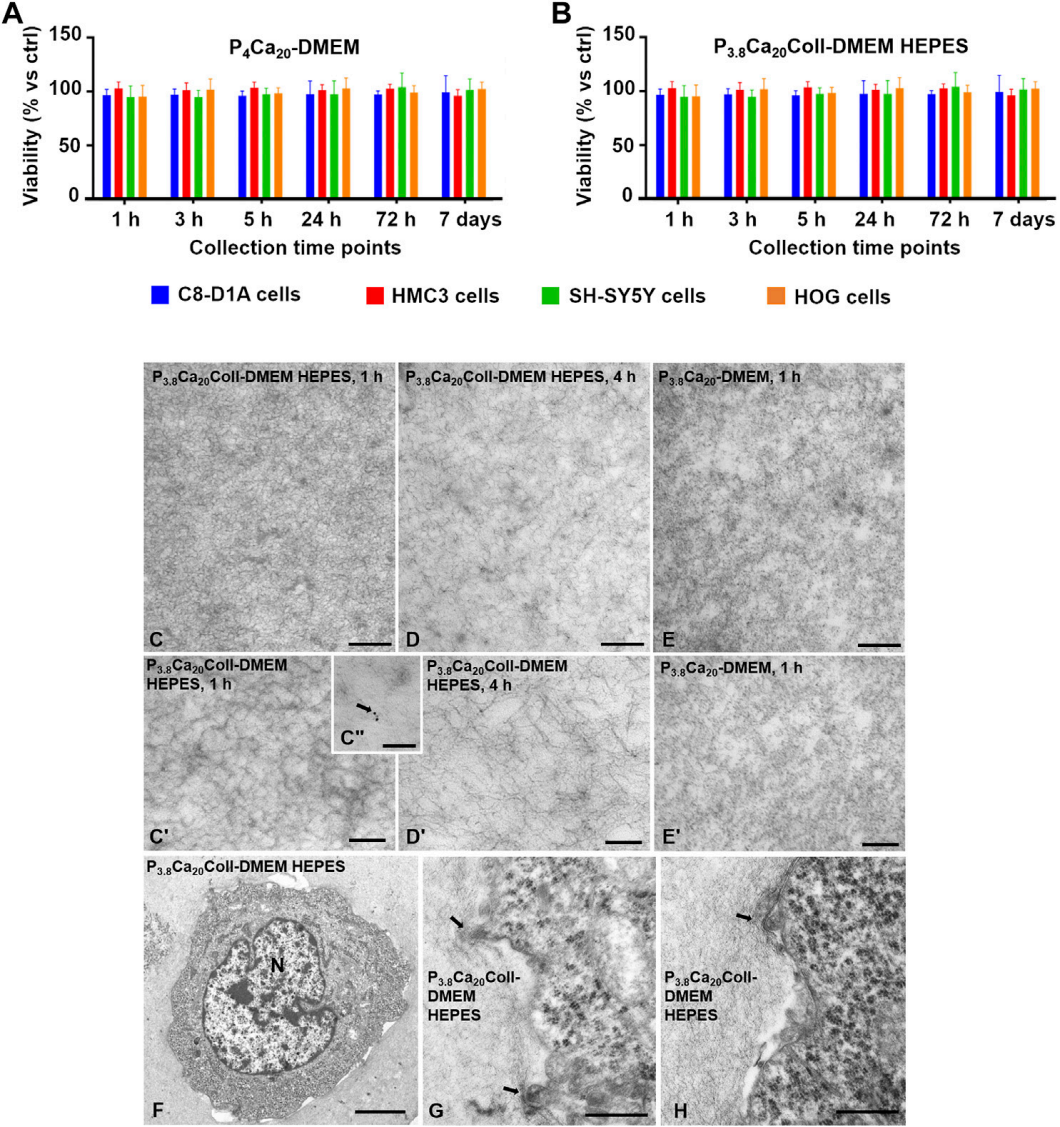
Viability of C8-D1A, HMC3, SH-SY5Y and HOG cells after 24 h in the supernatants collected after 1, 3, 5, 24, 72 h, and 7 days from P4Ca_20_-DMEM **(A)**, and P_3.8_Ca_20_Coll-DMEM HEPES **(B)** hydrogels. As a control, cells were grown for 24 h in fresh culture medium. Results from the MTS assay. Mean ± SD, 12 replicates/group. Data was analyzed with one-way ANOVA followed by Tukey’s multiple comparisons test. All the differences were not statistically significant (*p*-value >0.05). TEM images of P_3.8_Ca_20_Coll-DMEM HEPES after 1 h **(C, C′)** and 4 h **(D, D′)** crosslinking, and P_3.8_Ca_20_-DMEM after 1 h **(E, E′)** crosslinking, showing that in P_3.8_Ca_20_Coll-DMEM HEPES, collagen fibers were crosslinked together forming a dense network, while in P_3.8_Ca_20_-DMEM a uniform-looking matrix was present. Immunogold assays highlighted the specific localizations of gold particles associated to collagen fibers (arrow in Cʺ). C8-D1A astrocytic-like cells seeded in P_3.8_Ca_20_Coll-DMEM HEPES **(F)** showed contact and interacted with collagen fibers (arrows in **(G, H)**). N, nucleus. Bars in C-E, G, and H are 0.5 µm; bars in C′ and D′ are 200 nm; bar in Cʺ is 100 nm; bar in F is 5 µm.

The effect of both pectin and collagen concentrations on cell viability was investigated by culturing the cells for 24 h in the supernatants from P_4_Ca_20_-DMEM (Figure 8A) or P_3.8_Ca_20_Coll-DMEM HEPES (Figure 8B). As they were not specifically developed for cell encapsulation, we neglected 0.9% w/v NaCl-based compositions and tested DMEM-based formulations. We considered a pectin concentration of 4% w/v because it is the highest that has been tested in this study, and the pH values in the first crosslinking phases were than the ones for P_3.8_Ca_20_-DMEM (Figure 2). We hypothesized that P_4_Ca_20_-DMEM is cytocompatible with SH-SY5Y, C8-D1A, HMC3, and HOG cells, any similar pectin gel is expected to be too, but with a lower pectin concentration. For all the collection times and cell populations, viability, which was evaluated by MTS, was comparable with controls in standard medium (*p*-value>0.05). Although the crosslinking reaction is still not complete, culture medium can be immediately added to cover the samples and its addiction speeds up the reaching of pH values compatible with cell survival. The selected collagen content did not reduce cell viability. Again, for all the cell populations, viability was comparable with controls for all the time points (*p*-value>0.05).

### 3.4 Microstructural characterization: Transmission electron microscopy

Ultrastructural analysis by TEM of cell-embedding samples fixed after 60 min (Figures 8C,C') or 4 h culture (Figures 8D,D') showed that collagen fibers enriching pectin matrix in P_3.8_Ca_20_Coll-DMEM HEPES were crosslinked together forming a dense network. The immunogold assay (Figure 8C'') confirmed the specific localization of the gold-conjugated secondary antibody associated with collagen fibers at both 60 min and 4 h (arrow in Cʺ). In contrast, a loose and uniform-looking matrix was observed in P_3.8_Ca_20_-DMEM, in which collagen fibers were absent (Figures 8E,E'). In P_3.8_Ca_20_Coll-DMEM HEPES embedding C8-D1A astrocytic-like cells (Figure 8F), the matrix surrounding the cells appeared more compact and denser (Figures 8F–H).

### 3.5 Direct cytocompatibility after 3D-bioprinting

**FIGURE 9:**
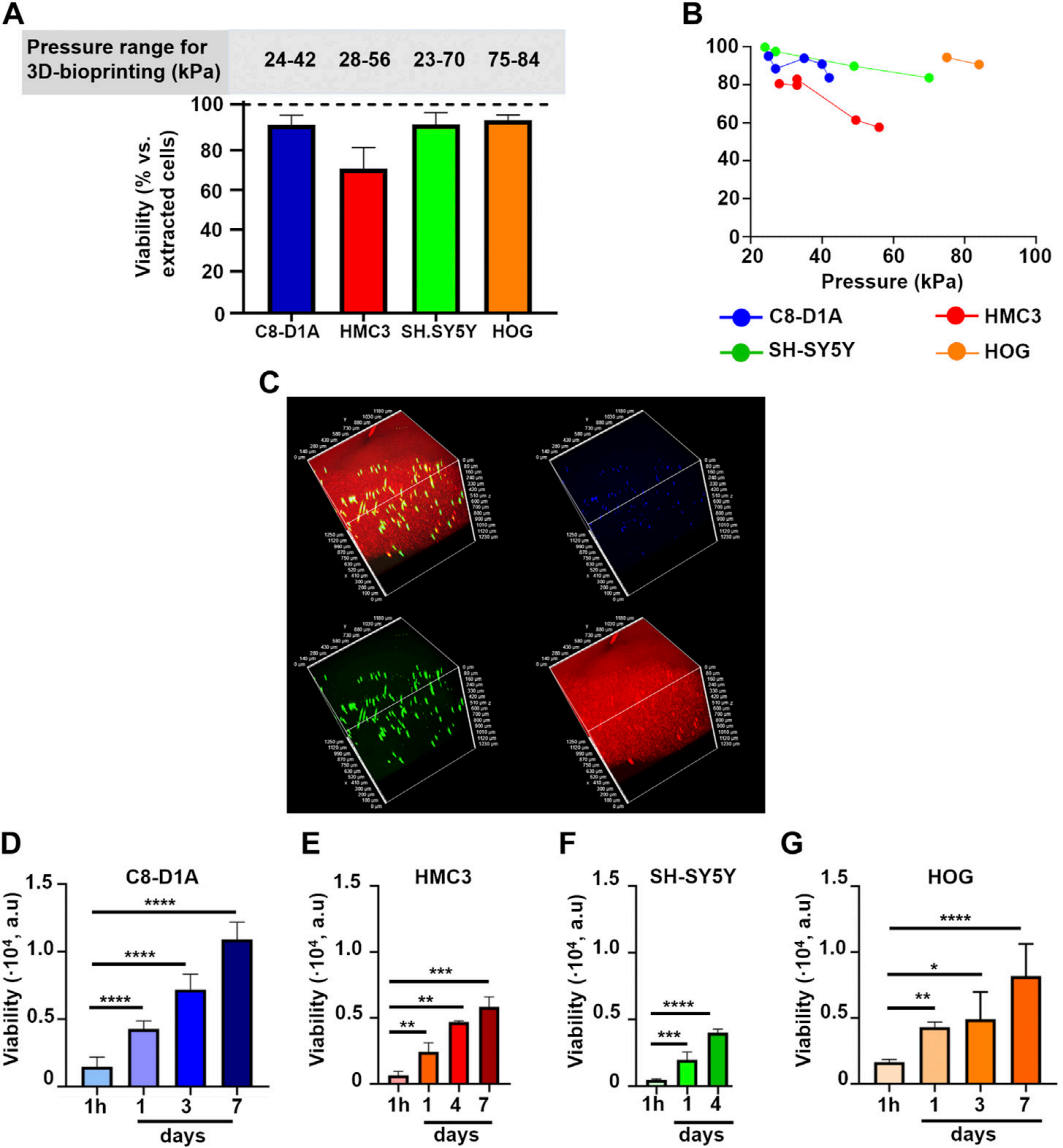
**(A)** Percentage of live cells with respect to the total number of counted cells after dissociating P_3.8_Ca_20_Coll-DMEM HEPES constructs. Results from the trypan blue exclusion assay for C8-D1A, HMC3, SH-SY5Y, and HOG cells. Mean ± SD, at least six replicates/condition. The upper panel shows the pressure ranges to print the constructs **(B)** Percentage of live cells with respect to the total number of counted cells after dissociating P_3.8_Ca_20_Coll-DMEM HEPES constructs as a function of printing pressure. Each point on the graph represents the average of three constructs printed consecutively. Results from the trypan blue exclusion assay for C8-D1A, HMC3, SH-SY5Y, and HOG cells **(C)** 3D reconstruction of C8-D1A cells 3D-bioprinted in P_3.8_Ca_20_Coll-DMEM HEPES. Viable cells were stained green, while cell nuclei were stained blue. P_3.8_Ca_20_Coll-DMEM HEPES was stained red, because it trapped the ethidium bromide fluorescent probe (Rounds et al., 2011); Viability of C8-D1A **(D)**, HMC3 **(E)**, SH-SY5Y **(F)**, and HOG **(G)** cells printed in P_3.8_Ca_20_Coll-DMEM HEPES inks and cultured over time. Results from the resazurin-based assay. Mean ± SD, at least 4 replicates/condition. Data was analyzed with one-way ANOVA followed by Tukey’s multiple comparisons test. ****: *p*-value <0.0001; ***: *p*-value <0.001; **: *p*-value <0.01; and *: *p*-value <0.05.

A necessary condition to support cell proliferation in P_3.8_Ca_20_Coll-DMEM HEPES is that cells survive the embedding process and the extrusion-based 3D-bioprinting with different cell types (SH-SY5Y human neuroblastoma cells, C8-D1A mouse astrocytes, HMC3 human microglial cells, and HOG human oligodendroglioma cells). For this reason, we evaluated cell viability immediately (t ≤ 60 min) after bioprinting. Cell viability (i.e. the number of live cells with respect to the total number of counted cells, Figure 9A) was similar for HOG cells, SH-SY5Y cells, C8-D1A cells, and slightly lower for HMC3 cells ((73 ± 10) % vs. ∼90%). Cell counts were carried out after dissolving the constructs with sodium citrate, a Ca^2+^ chelating agent with improved cytocompatibility with respect to ethylenediaminetetraacetic acid (EDTA, Amaral et al., 2007). To speed up the disaggregation process, we coupled chemical and mechanical actions by pipetting sodium citrate on the constructs for a few minutes. As the process required manipulations after embedding and 3D-bioprinting, our results underestimated cell viability. The fact that cell viability was always comparable with controls supports the potential of P_3.8_Ca_20_Coll-DMEM HEPES for the 3D-bioprinting of neural cells.

Although derived from pectin solutions in the same pH range, cell constructs were printed at different pressures (Figure 9A), sometimes greater than those for cell-free samples. We observed that the presence of cells could influence the viscoelastic properties (data not shown). For instance, hydrogels with SH-SY5Y and HMC3 cells shared comparable crosslinking kinetics, with minimal changes of viscoelastic properties over time with respect to their cell-free counterparts. After 40 min crosslinking, for samples with SH-SY5Y cells, *Gʹ* and *G*ʺ reduced by 5% and 3%, while for samples with HMC3 cells, *Gʹ* and *Gʺ* decreased by 7% and 2%. On the contrary, C8-D1A cells acted as a reinforcement for P_3.8_Ca_20_Coll−DMEM HEPES. *Gʹ* and *Gʺ* increased by 51%, and 41% with respect to cell-free samples, respectively (data not shown).

Cell-laden P_3.8_Ca_20_Coll-DMEM HEPES maintained the shear-thinning behavior, but we observed an increase in viscosity values over crosslinking time. The presence of cells reduced the ability of the material to be extruded through the nozzle, and required an increase in printing pressure. In addition, cells were embedded at high densities. To obtain a homogeneous cell dispersion and avoid aggregation, frequent mixing phases were required, leading to the formation of air bubbles. We speculate that it could explain the high pressures required for HOG-laden constructs. Indeed, visual inspections suggested that HOG cells were the biggest used in this study and thus potentially subjected to a fast sedimentation within the polymer solutions.

Since multiple grids can be printed from the same cartridge, we exploited the results in Figure 9A to investigate whether the increasing pressure could compromise cell survival (Figure 9B). For all the cell populations, the first set of scaffolds showed the highest viability, but a reduction was observed with increasing pressure (i.e. over time). For instance, about 100% of SH-SY5Y cells in the first three constructs (printed at 23 kPa) were viable, but viability dropped to 84% when pressure was increased to 60 kPa. On the contrary, the viability of HMC3 cells showed a drastic decrease after the second set of grids (from 81% at 28 kPa to 58% at 56 kPa).

Sample observation by confocal microscopy (Figure 9C) highlighted that the printing protocol did not affect cell viability, also showing that cells were homogenously distributed within the inks. In fact, they were visible in the green channel, meaning that they internalized the calcein-AM probe. The red background could be explained by ethidium homodimer-1 (sharing structural similarities with propidium iodide), which competed with Ca^2+^ in labelling pectin, as results from the literature (Rounds et al., 2011).

To strengthen the results from the trypan blue exclusion assay, viability after 3D-bioprinting was evaluated for all cell populations by a resazurin-based assay after 1 h, 1, 3 (or 4), and eventually 7 days of culture (Figures 9D–G). For all cell populations, viability increased over time. After embedding and 3D-bioprinting in P_3.8_Ca_20_Coll-DMEM HEPES, cells survived and kept their proliferative potential, suggesting that the proposed ink could be suitable to print and culture neural cells.

## 4 Discussion

Pectin and calcium concentrations tested in this study allowed to obtain 3D hydrogels mimicking the basic viscoelastic properties of brain tissue. In fact, at the end of time sweep scans, the elastic moduli of both gels in 0.9% w/v NaCl and DMEM w/and w/o HEPES fell in the range reported for brain tissue (i.e. from few hundreds of Pa to kPa, Leipzig and Shoichet, 2009; Axpe et al., 2020). P_2.4_Ca_35_-NaCl showed the highest viscoelastic properties (*G´* = 1.41 × 10^3^ Pa; *G″* = 0.27 × 10^3^ Pa), with tanδ being about 0.2 (i.e. *G″* being about 20% *G′*), as is often found in physiological tissues (Charrier et al., 2018). All the other hydrogels also fulfilled condition 0.1 < tanδ < 0.2 (i.e. 10% *G′* < *G″* < 20% *G′*). Also due to its composition, we exploited P_3.8_Ca_20_Coll-DMEM HEPES for the 3D-bioprinting of neural cells and showed that internally crosslinked pectin-based hydrogels could be suitable for neural cell culture.

This work demonstrates that pectin-based inks produced by pre-crosslinking by internal gelation allow to fabricate self-standing fibers and multi-layer grids with a defined shape after extrusion. Additives or post-printing treatments were shown to be not required. Ink development was guided by the effects of pH and pectin concentration on the viscosity of solutions, together with the impact of the amount of the crosslinker, i.e. the content of calcium salts, on the viscoelastic properties and printability of the gels. Since solvent composition is fundamental to develop a printable ink, we decided to increase complexity to catch up common basic elements and speed up the optimization. Firstly, we tested pectin solutions in 0.9% w/v NaCl, then solutions in DMEM with additives and finally in DMEM also supplemented with HEPES. Our method exploited rheology to give *a priori* information about ink printability and it coupled pH and printing time to gain full control over the kinetics of internal crosslinking. By introducing the pH of pectin solutions as a further parameter to be controlled, we were able to have multiple (pH-dependent) crosslinking kinetics without varying hydrogel composition. Basically, we added a tuning parameter to the time and expanded the potential of the previously described 3D-reactive printing strategy (Sardelli et al., 2021). This opens to the possibility of applying this approach also to materials whose crosslinking kinetics depended on the pH. Overall, whatever the parameter that controls the crosslinking kinetics, it is possible to exploit it to modulate the printing time, i.e. the stage of the crosslinking in which a hydrogel in formation can be printed. In our case, this was strategic to minimize the persistence time of the cells in the ink before printing, while still allowing for a proper viscosity control.

The content of the crosslinking agent, i.e. CaCO_3_ as a source of Ca^2+^, was also a key parameter to determine the presence of insoluble deposits, fiber quality, and printing time. Pectin solutions at a concentration of 2.4% w/v in 0.9% w/v NaCl showed an acidic pH (3.4 ± 0.1), which was able to trigger a fast gelation even when mixed to the lower CaCO_3_ concentration (20 mM). This concentration also allowed to avoid calcium deposits in printed fibers. In contrast, the higher concentration (35 mM CaCO_3_) resulted in high pressures for extrusion-based 3D-printing (Figure 4). An increase in CaCO_3_, that correlates with enhanced hydrogel stiffness and reduced diffusion, may decrease cell viability and proliferation (Banerjee et al., 2009; Ahn et al., 2012). Thus, we produced internal crosslinking with 20 mM CaCO_3_.

Keeping in mind both 3D-printing and cellular studies, we controlled ink compositions to tune their properties. More specifically, starting from the hypothesis that solutions with comparable viscosity profiles exhibit similar printability in the presence of the same amount of CaCO_3_, we identified a suitable pectin concentration to reproduce the flow curve for the printable 2.4% w/v pectin in 0.9% w/v NaCl including a cell culture medium (DMEM). Moreover, to mimic common cell culture conditions, we enriched basal DMEM with serum, l-glutamine and antibiotics. The buffering capacity of DMEM was required to increase pectin concentration to 4% w/v to meet similar values of viscosity. The change from 0.9% w/v NaCl to DMEM speeded up the crosslinking kinetics in the early phase (20 min) and increased *G″* (Figures 2C, D). In the recovery curves at 0 min (Supplementary Figures S1A, B), *G″* values were greater for the ink produced in DMEM than in NaCl, but after 30 and 60 min crosslinking, the recovery trends of *G″* were similar. The shift to DMEM also impacted extrudability (Supplementary Figures S1C–E) at time 0, when P_2.4_Ca_20_-NaCl could be extruded more easily than P_4_Ca_20_-DMEM. At 30 and 60 min, extrudability depended on the shear rate: for shear rates lower than 10 s^−1^, the extrusion of DMEM ink was easier than the one of the NaCl ink; for higher shear rates, the opposite occurred. The comparison between the printability windows of P_2.4_Ca_20_-NaCl and P_4_Ca_20_-DMEM highlighted the effect of *G″*: after 30 and 60 min, the maximum pressures to extrude P_4_Ca_20_-DMEM were like the ones for P_2.4_Ca_35_-NaCl, but at 0 min they were even greater, and thus excessive for extrusion-based 3D-bioprinting.

To reduce hydrogel *G″* without remarkable impact on the crosslinking kinetics, we decreased pectin concentration to 3.8% w/v without varying pH (3.5 ± 0.1) and produced P_3.8_Ca_20_-DMEM. The shift also reduced the maximum pressures for extrusion to values like the ones for P_2.4_Ca_20_-NaCl.

But still, we needed to optimize the produced bioink for cell cultures by acting on the pH in the early stages of crosslinking. Towards this aim, the CO_2_/bicarbonate system already in basal DMEM (44 mM NaHCO_3_) was coupled with HEPES. This supplementation did not vary the viscosity of the solution (Supplementary Figure S1F). To improve the cell adhesion properties of pectin (Chen et al., 2018) with a strategy simpler and cheaper than grafting with motif peptides, cells were loaded into collagen solutions at neutral pH and then mixed to pectin/CaCO_3_ 3 min after the beginning of crosslinking. With this protocol, cells were loaded into pectin gels with pH around 5.81. Cell culture medium was immediately added to speed up the reaching of physiological pHs. For this reason, P_3.8_Ca_20_Coll-DMEM HEPES was selected for 3D-bioprinting. For its preparation, solutions with 3.55 < pH < 3.70 were used. However, even small pH variations have a remarkable effect on the viscoelastic properties of the final gels (Supplementary Figure S1G). When pH increased from 3.55 to 3.75, *G′* and *G″* decrease. After 60 min crosslinking, for solutions with 3.55 < pH < 3.60, *G′* and *G″* reached 661 Pa and 73 Pa, respectively. For solutions with 3.71 < pH < 3.75, *G′* and *G″* reduced to 213 Pa and 43 Pa, respectively. For pH > 3.79, gelation did not occur in 1 h. The pH of pectin solutions was always measured prior the experiments because it was shown to also influence the crosslinking kinetics and the resting time (i.e. the time before printing constructs with uniform fibers and open porosities): the higher the pH was, the slower was the crosslinking kinetics and the longer the resting time before printing self-standing fibers. This needed to be considered to optimize the viscosity, the printing velocity, and the printing pressure.

Finally, our study proposes an innovative way of exploiting pectin/collagen combinations. Interactions based on surface patch binding were described in collagen/pectin composites loaded with bioactive glass nanoparticles (Wenpo et al., 2015; Goel et al., 2021). As results from previous works, pectin and collagen exhibit the same net charge, but the positively charged patches on collagen bind to the negatively charged segments on pectin, and Ca^2+^ acts as a bridge between—C=O and—COO groups (Goel et al., 2021). When neutral pectin/collagen solutions are combined (Jayakumar et al., 2014), the basic amino acids of collagen primarily interact with pectin, that stabilizes collagen by hydrogen bonding. It creates an effective defense mechanism against collagenase, and promotes collagen stability. Interestingly, an increase in pectin concentration (i.e. in the number of interaction sites) limits the mobility of collagen molecules, leading to the formation of collagen fibrils in the ordered form of precipitates. The mixing in neutral conditions of alkaline de-esterified pectin/CaCl2 with different rations of collagen type I and/or IV (and the subsequent incubation at 37°C) was reported to create an optimal microenvironment for glioblastoma treatment (Belousov et al., 2020).

With these results in mind, together with the possibility of using our inks both for the realization of *in vitro* 3D models of neural tissue, and for cell delivery in clinical practice (e.g. stroke), we deeply focused on ink development and cell encapsulation. The exploitation of pectin as the bulk material provides an extremely varied skeleton on which it is potentially possible to graft molecules and signals to modulate cell behavior. The change from external to internal crosslinking allowed to achieve homogeneous hydrogels, with advantages in terms of providing uniform stimuli for cell culture. By mixing neutral collagen with a high pectin concentration, we speeded up collagen aggregation, and removed the need for incubation at 37°C. The proposed ink was suitable to produce multiple stacks of grids by 3D-printing, and promoted the adhesion of cells to the matrix, favoring their viability over time. Not only cells survived and proliferated after 3D-bioprinting, but they also interacted with the ink, as suggested by the time sweeps of cell-laden constructs. In addition, the structural reorganization of the hydrogels observed by TEM was probably cell-mediated and achieved thanks to the interaction between the matrix and the cell membranes (Figures 8F–H), leading to a change in the orientation of collagen fibers (Webber et al., 2016; Lovrak et al., 2017). Finally, with respect to clinical applications, a strict control over the pH of pectin solution allowed to tune the crosslinking kinetics, i.e. the time available to the ink to conform to the defect to be filled.

## Data Availability

The original contributions presented in the study are included in the article/Supplementary Material, further inquiries can be directed to the corresponding author.
