# Supplementary material for: Pectin-based bioinks for 3D models of neural tissue produced by a pH-controlled kinetics
Source: Front Bioeng Biotechnol. 2022 Dec 22;10:1032542. doi: 10.3389/fbioe.2022.1032542 (PMC9815771; doi:10.3389/fbioe.2022.1032542)
Supplement: Supplementary file 1 [file DataSheet1.PDF]

Supplementary Material

Supplementary Table 1: Flow behavior at 60 min. It was interpreted in terms of a power law relationship

**HYDROGELS**	***K***	***n***
$P_{2.4}Ca_{20}$-NaCl	$22.90 \cdot 10^{4}$	$8.65 \cdot 10^{-3}$
$P_{2.4}Ca_{35}$-NaCl	$6.50 \cdot 10^{4}$	$7.80 \cdot 10^{-3}$
$P_{4}Ca_{20}$-DMEM	$15.01 \cdot 10^{4}$	$1.43 \cdot 10^{-1}$
$P_{3.8}Ca_{20}$-DMEM	$10.42 \cdot 10^{4}$	$1.11 \cdot 10^{-1}$
$P_{3.8}Ca_{20}$Coll-DMEM HEPES	$8.39 \cdot 10^{4}$	$1.91 \cdot 10^{-1}$

Supplementary Table 2: Diameters of $P_{2.4}Ca_{20}$-NaCl, $P_{3.8}Ca_{20}$-DMEM, and $P_{3.8}Ca_{20}$Coll-DMEM HEPES single fibers printed at minimum pressures. Mean ± SD, 6 replicates/condition

Ink	Nozzle (µm)	Speed (mm/s)	Time (min)	Fiber diameter (µm)
**$P_{2.4}Ca_{20}$-NaCl**	410	10	0	792 ± 94
			30	669 ± 98
			60	890 ± 124
		15	0	474 ± 48
			30	1047 ± 179
			60	658 ± 58
	250	10	0	474 ± 99
			30	579 ± 106
			60	903 ± 178
		15	0	205 ± 17
			30	209 ± 12
			60	202 ± 36
**$P_{3.8}Ca_{20}$-DMEM**	250	10	0	712 ± 142
			30	806 ± 152
		15	0	450 ± 40
			30	989 ± 129
**$P_{3.8}Ca_{20}$Coll-DMEM HEPES**	250	10	0	603 ± 37
			30	641 ± 72
		15	0	789 ± 78
			30	637 ± 74

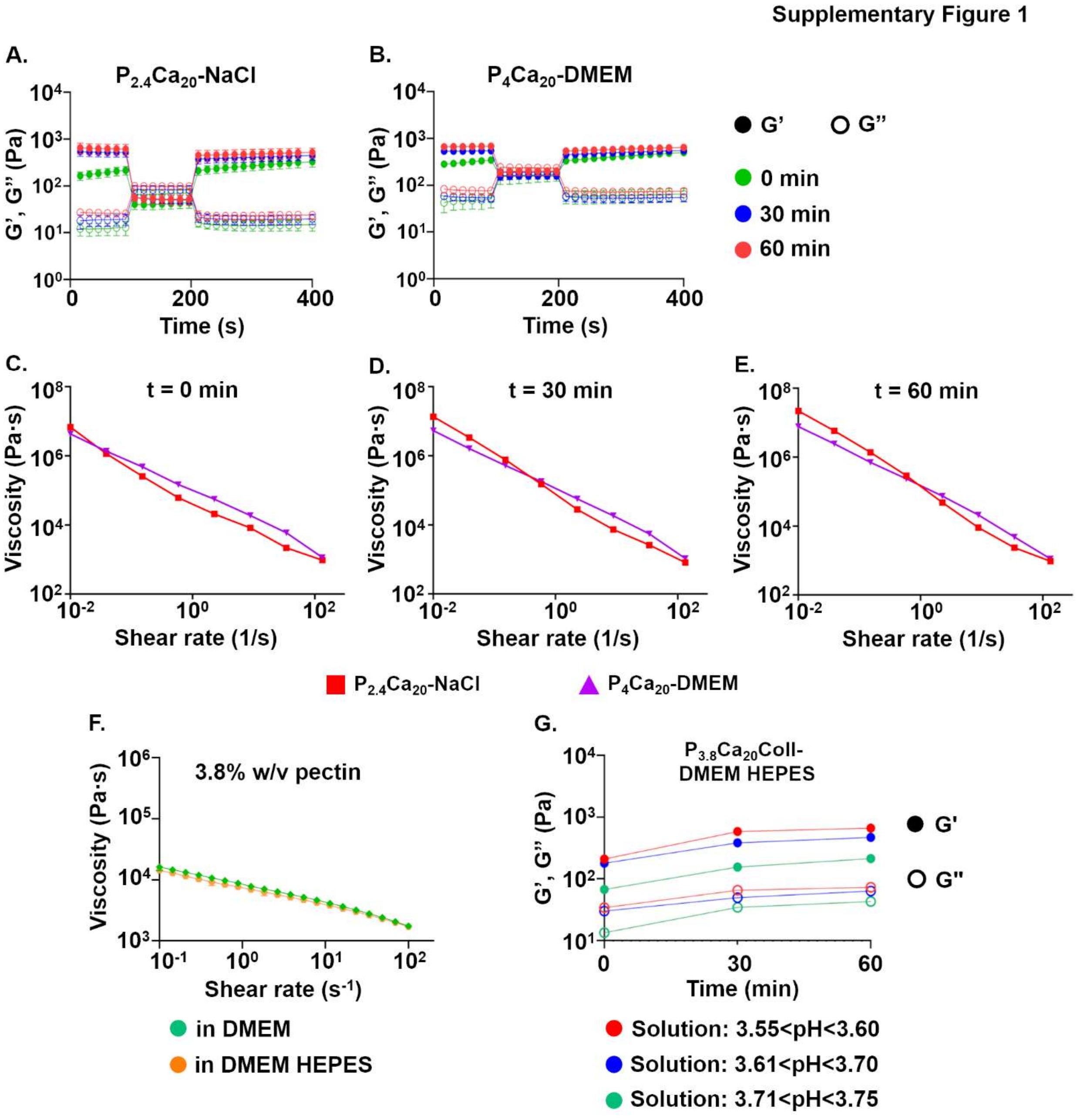

Supplementary Figure 1. Recovery of *G′, G″* as a function of test time for $P_{2.4}Ca_{20}$-NaCl (A) and P_4Ca_{20}-DMEM (B) after applying a 100% strain at 1 Hz for 100 s. Mean ± SD, 4 replicates/condition; Flow curves for $P_{2.4}Ca_{20}$-NaCl and P_4Ca_{20}-DMEM at 0 (C), 30 (D) and 60 min (E). Mean ± SD, 4 replicates/condition; F) Flow curves for 3.8% w/v pectin in DMEM w/ or w/o HEPES. Mean ± SD, 12 replicates/condition. G) *G′* and *G″* over crosslinking time as a function of the pH of the initial pectin solution for $P_{3.8}Ca_{20}$Coll-DMEM HEPES, Mean values, 3 replicates/condition.
